# Nasal delivery of single-domain antibody improves symptoms of SARS-CoV-2 infection in an animal model

**DOI:** 10.1371/journal.ppat.1009542

**Published:** 2021-10-14

**Authors:** Kei Haga, Reiko Takai-Todaka, Yuta Matsumura, Chihong Song, Tomomi Takano, Takuto Tojo, Atsushi Nagami, Yuki Ishida, Hidekazu Masaki, Masayuki Tsuchiya, Toshiki Ebisudani, Shinya Sugimoto, Toshiro Sato, Hiroyuki Yasuda, Koichi Fukunaga, Akihito Sawada, Naoto Nemoto, Kazuyoshi Murata, Takuya Morimoto, Kazuhiko Katayama

**Affiliations:** 1 Laboratory of Viral Infection, Department of Infection Control and Immunology, Ōmura Satoshi Memorial Institute & Graduate School of Infection Control Sciences, Kitasato University, Tokyo, Japan; 2 Safety Science Laboratories, Kao Corporation, Tokyo, Japan; 3 Exploratory Research Center on Life and Living Systems (ExCELLS), National Institutes of Natural Sciences, Okazaki, Japan; 4 National Institute for Physiological Sciences, Okazaki, Japan; 5 School of Veterinary Medicine, Kitasato University, Towada, Japan; 6 Biological Science Laboratories, Kao Corporation, Wakayama, Japan; 7 Epsilon Molecular Engineering Inc., Saitama, Japan; 8 Department of Organoid Medicine, Keio University School of Medicine, Tokyo, Japan; 9 Department of Pulmonary Medicine, Keio University School of Medicine, Tokyo, Japan; Icahn School of Medicine at Mount Sinai, UNITED STATES

## Abstract

The severe acute respiratory syndrome coronavirus 2 (SARS-CoV-2) that causes the disease COVID-19 can lead to serious symptoms, such as severe pneumonia, in the elderly and those with underlying medical conditions. While vaccines are now available, they do not work for everyone and therapeutic drugs are still needed, particularly for treating life-threatening conditions. Here, we showed nasal delivery of a new, unmodified camelid single-domain antibody (VHH), termed K-874A, effectively inhibited SARS-CoV-2 titers in infected lungs of Syrian hamsters without causing weight loss and cytokine induction. *In vitro* studies demonstrated that K-874A neutralized SARS-CoV-2 in both VeroE6/TMPRSS2 and human lung-derived alveolar organoid cells. Unlike other drug candidates, K-874A blocks viral membrane fusion rather than viral attachment. Cryo-electron microscopy revealed K-874A bound between the receptor binding domain and N-terminal domain of the virus S protein. Further, infected cells treated with K-874A produced fewer virus progeny that were less infective. We propose that direct administration of K-874A to the lung could be a new treatment for preventing the reinfection of amplified virus in COVID-19 patients.

## Author summary

Vaccines for COVID-19 are now available, but therapeutic drugs are still needed to treat life-threatening cases and those who cannot be vaccinated. We discovered a new heavy-chain single-domain antibody that can effectively neutralize the severe acute respiratory syndrome coronavirus 2 (SARS-CoV-2) that causes COVID-19. Unlike other drug candidates, which prevent the virus from attaching to the receptor on the host cell, this new antibody acts by blocking the virus membrane from fusing with the host cell membrane. We studied the behavior of the new antibody *in vitro* in VeroE6/TMPRSS2 cells and human lung organoids. When delivered through the nose to infected Syrian hamsters, this antibody prevented the typical symptoms caused by SARS-CoV-2. Our results are significant because delivering simple drugs directly to infected lungs with a nebulizer could increase the potency of the drugs while reducing the risk of immune reaction that could occur if the drugs escape or are delivered through the blood stream.

## Introduction

Coronaviruses (CoV) are enveloped, single-stranded positive-sense RNA viruses. They are divided into four genera: alpha, beta, gamma and delta. Betacoronaviruses are significant interest because they are responsible for the severe acute respiratory syndrome (SARS) and middle eastern respiratory syndrome (MERS) epidemics in the past and now, the coronavirus disease 2019 (COVID-19) pandemic. These viruses can cause mild to severe respiratory tract infections and death in some cases. All betacoronaviruses feature a spike protein on their surface that assists entry into cells. The spike (S) protein has two distinct subunits, S1 and S2, and the receptor binding domain (RBD) in the S1 subunit interacts with the host cell receptor. The S proteins of SARS-CoV-1 (virus causing SARS) and SARS-CoV-2 (virus causing COVID-19) bind to the same host cell receptor, angiotensin-converting enzyme 2 (ACE2). After binding to ACE2 via the RBD, a protease on the host cell surface cleaves and activates the S protein, allowing the virus membrane to fuse with the host cell membrane. Blocking this viral fusion is thought to be a promising therapeutic strategy [1,2]. Furthermore, because SARS-CoV-2 infects cells in the nasal mucosa or lungs that express ACE2 [3], direct delivery of antiviral drugs to the respiratory system is expected to improve drug efficacy.

Camelid single-domain antibodies are a unique class of antibodies that consist of a single heavy chain. Variable domain of heavy chain of heavy chain antibodies (VHHs) binds viruses, such as influenza virus, human immunodeficiency virus 1 (HIV-1), and respiratory syncytial virus (RSV) [4]. These antibodies, which consist of single amino acid chains, bind specific antigens with high affinity and specificity. Unlike human monoclonal antibodies, VHHs can be easily modified and produced using bacteria. They are also stable against heat and pH, allowing them to be stored longer than human monoclonal antibodies and, therefore, stockpiled for epidemics. Being stable also means VHHs can be nebulized and administered via an inhaler directly to infected lungs. The first VHH undergoing clinical trials for direct delivery into the lung by nebulization to treat RSV was ALX-0171 [4]. *In vitro* studies show ALX-0171 binds to the F protein in RSV with higher affinity than the approved human monoclonal antibody prophylactic drug, Palivizumab [5]. Intranasal administration to cotton rats reduced RSV load in the nose and lung [5,6]. Recently, a VHH or single-domain antibody against SARS-CoV-1 or SARS-CoV-2 has also been identified [7–12], and these VHHs inhibit ACE2 binding and block the infection. The VHH (termed VHH-72) neutralizes SARS-CoV-1; however, it must be fused with an Fc domain of a human antibody to neutralize SARS-CoV-2 [7]. Fc-fusion enhanced the neutralizing effect of Ty1 [9], Sb23 [10], and H11-H4 [11], but not that of n3088, n3130 [8] and Nb11-59 [12]. While effective in neutralizing the virus *in vitro*, exactly it remains unclear how these VHHs perform *in vivo*.

Here, using the S1 domain of SARS-CoV-2 S protein as an antigen, we screened an extensive DNA library and found a standalone VHH that is specific for SARS-CoV-2. This new VHH (termed K-874A) bound SARS-CoV-2 S protein with higher affinity than previous VHHs [7,8] and does not require any modification with antibody fragments, making them a very attractive therapeutic candidate. We showed that K-874A effectively neutralizes SARS-CoV-2 *in vitro*. When intranasally administered to SARS-CoV-2-infected Syrian hamsters, K-874A prevented weight loss, reduced viral replication in the lungs and prevented cytokine induction that are characteristic of a severe SARS-CoV-2 infection. Our results demonstrated the K-874A may lead to develop therapeutic drug against SARS-CoV-2.

## Results

### *In vitro* selection of anti-SARS-CoV-2 S1 VHHs

**Fig 1:**
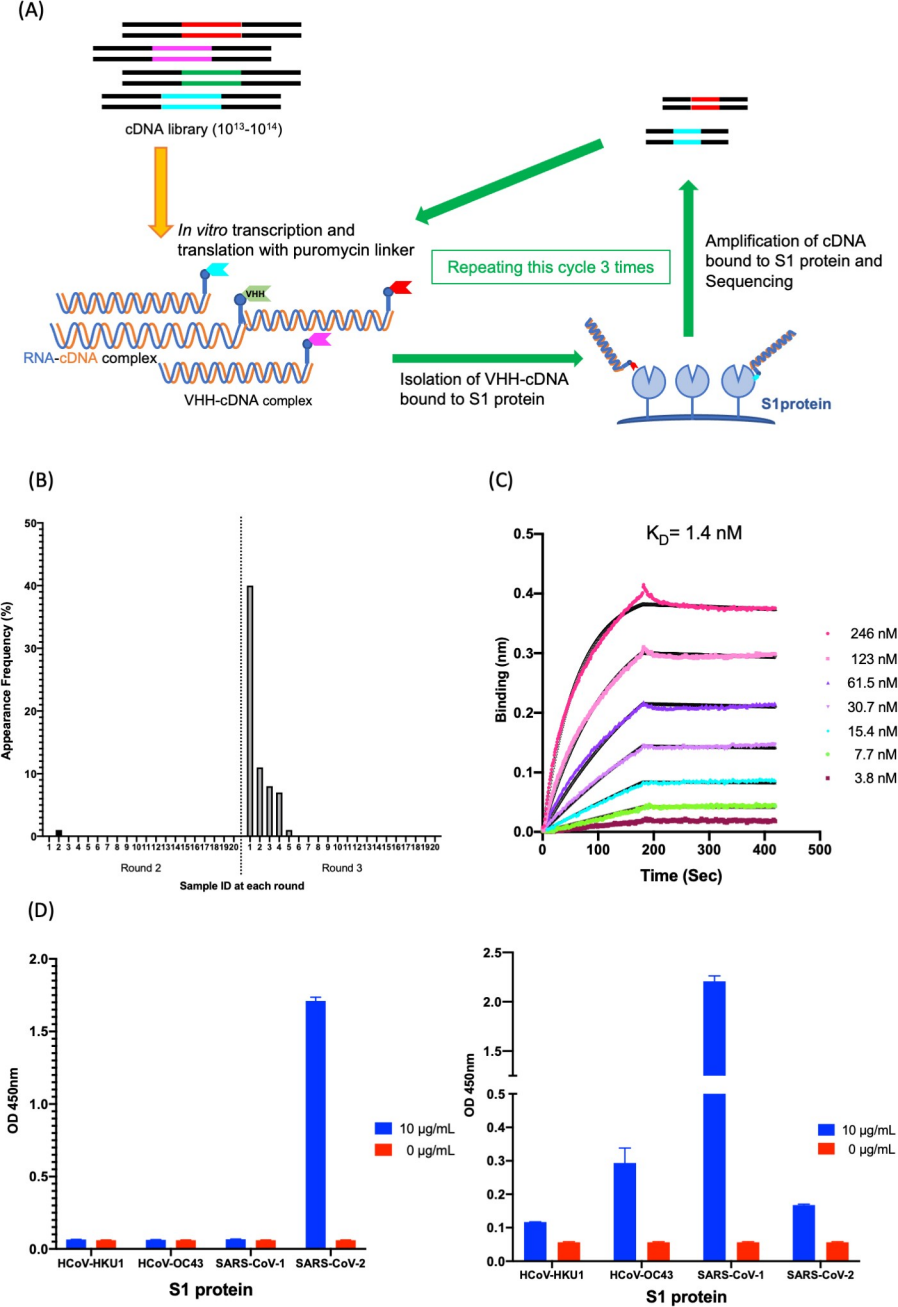
Isolation and characterization of K-874A. (**A**) Schematic showing *in vitro* selection of VHHs against SARS-CoV-2 S1 protein using VHH-cDNA display. *In vitro* transcription and translation of VHH-cDNA library form VHH linked to its mRNA with a puromycin linker. cDNA of linked mRNA was reverse-transcribed and VHH-cDNA complex was produced. High-affinity VHH-cDNA complex to immobilized S1 protein was isolated, and its cDNA was amplified. Three rounds of selection were performed, cDNA libraries from rounds 2 and 3 were sequenced, and anti-SARS-CoV-2 VHH candidates were translated. (**B**) Frequency distribution of amino acid sequences corresponding to VHH antibody candidates targeting SARS-CoV-2 S1 subunits in the selected VHH libraries. Sample ID “1” with the highest frequency (39.5%) is clone K-874A. (**C**) Binding affinity of K-874A to SARS-CoV-2 S1 subunits. Biolayer interferometry sensorgram measures the apparent binding affinity of K-874A-6xHis to immobilized SARS-CoV-2 S1 fused with sheep Fc. Binding curves for different concentrations of K-874A are shown in different colors. The black curves are 1:1 fits of the data. (**D**) Direct antigen ELISA measuring the binding affinity of FLAG-tagged K-874A to immobilized S1-6xHis subunits of beta-coronaviruses (HCoV-HKU1, HCoV-OC43, SARS-CoV-1, SARS-CoV-2) (left). Each immobilized S1-6xHis subunit was detected by anti-His antibody (right). Error bars are mean ± SD (N = 3). Data are from a representative experiment of three independent experiments.

We used VHH-cDNA display for *in vitro* selection of VHHs against the SARS-CoV-2 S1 protein (Fig 1A). cDNA display yields functional VHHs, whose coding RNA is linked via a puromycin linker [13–15]. This procedure selects the coding sequences for high-affinity VHHs from a diverse (10^13−14^) DNA library [16]. To select the VHH candidates targeting SARS-CoV-2 S1 proteins, DNA libraries at Round 2 (R2) and Round 3 (R3) of *in vitro* selection were sequenced, and anti-SARS-CoV-2 VHH candidates were translated.

From the frequency distribution of VHH clones in the selected library, clone K-874A appeared most frequently (39.5%) after three rounds of *in vitro* selection, indicating that K-874A has a high affinity for S1 proteins (Fig 1B). Biolayer interferometry assay showed the binding affinity of K-874A to SARS-CoV-2 S1 protein is 1.4 nM (K_a_ (1/Ms) = 6.72E+04, K_d_ (1/s) = 9.42E-05) (Fig 1C). When a direct antigen enzyme-linked immunosorbent assay (ELISA) was performed using FLAG-tagged K-874A and immobilized recombinant His-tagged S1 protein, we found that K-874A bound with high affinity to the S1 protein of SARS-CoV-2 but not to other coronaviruses, including human CoV (HCoV-HKU1, HCoV-OC43) and SARS-CoV-1, even when all S1-6xHis subunit were immobilized (Fig 1D). These results indicate that K-874A binds strongly and specifically to the S1 protein of SARS-CoV-2.

### K-874A VHH prevents SARS-CoV-2 fusion with host cell

**Fig 2:**
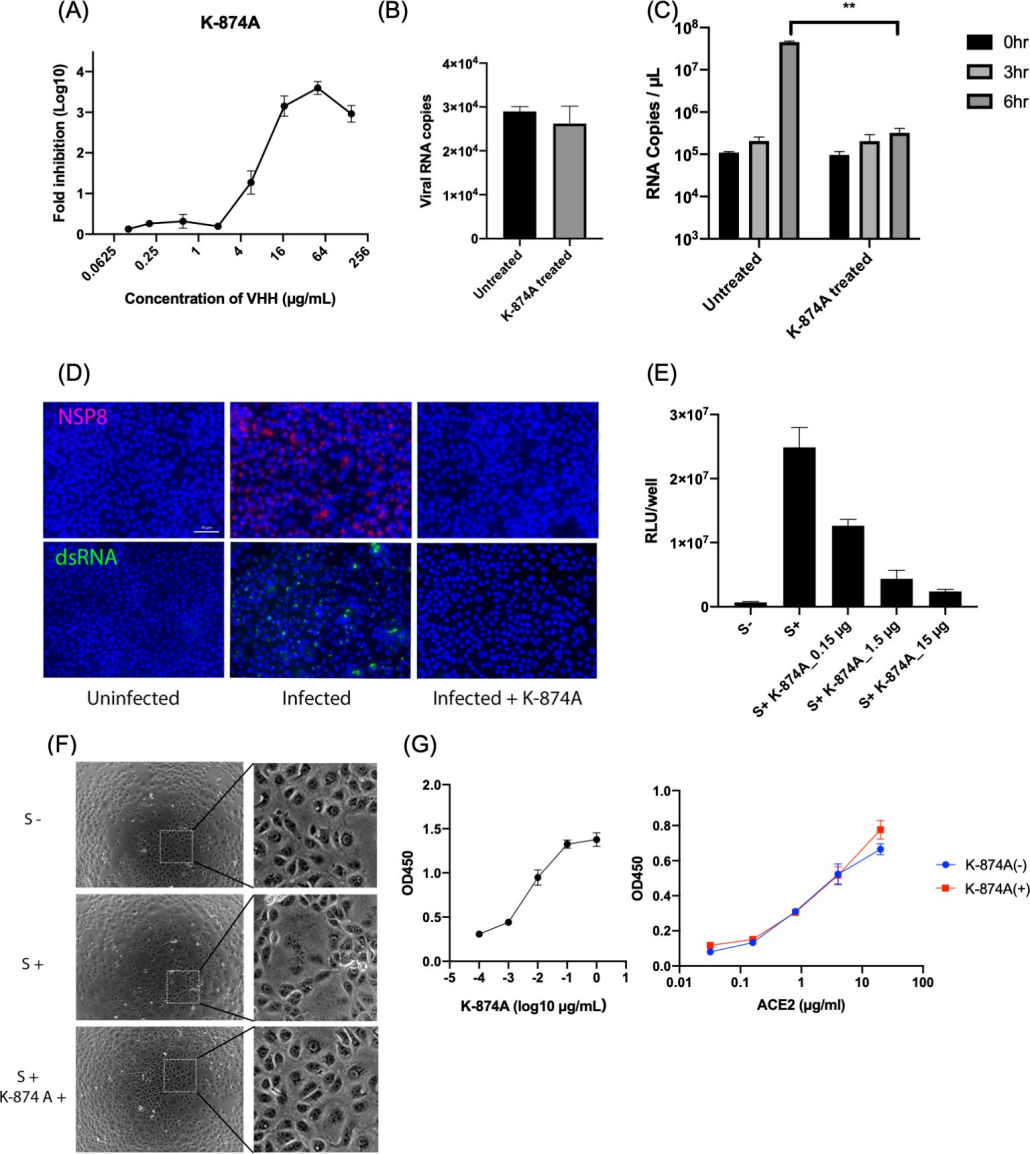
K-874A VHH neutralizes SARS-CoV-2 by preventing viral fusion with host cell. (**A**) Neutralization of SARS-CoV-2 by K-874A VHH. SARS-CoV-2 was pretreated with different concentrations of K-874A and inoculated into VeroE6/TRMPSS2 cells. Fold-reductions in viral RNA copies in culture supernatants were calculated by qRT-PCR. K-874A has an IC_50_ of 5.74±2.6 μg/ml. Error bars indicate mean ± SD from three independent experiments. (**B**) The numbers of viral RNA copies in VeroE6/TMRPSS2 cells infected with SARS-CoV-2 (untreated) and SARS-CoV-2 pretreated with K-874A VHH (K-874A-treated) were similar. Number of RNA copies attached or incorporated in the cells were determined by qRT-PCR. Error bars indicate mean ± SD from three independent experiments. (**C**) Number of viral RNA copies in VeroE6/TMRPSS2 cells infected with untreated SARS-CoV-2 and K-874A-treated SARS-CoV-2 at 0, 3 and 6 hr. RNA levels were estimated by qRT-PCR (N = 3, **P<0.005, Welch’s t test). (**D**) Immunofluorescence images show viral protein, NSP8, and RNA replication are suppressed in Vero/TMPRSS2 cells infected with K-874A-treated SARS-CoV-2. SARS-CoV-2-infected cells were fixed at 24 hr after infection and treated with anti-NSP8 (red) or anti-dsRNA (green). After fluorescence-conjugated secondary antibody and Hoechest (blue) treatment, images were captured by BZX800. Scale bar = 50 μm. (**E**) Cell fusion induced by S protein transduction in HiBiT- and LgBiT-expressing VeroE6/TMPRSS2 cells. Treatment with K-874A VHH suppressed cell fusion in a dose-dependent manner. Error bars indicate mean ± SD from three independent experiments. (**F**) Optical images of VeroE6/TMPRSS2 cells expressing S protein transduced by a lentivirus vector show cell fusion (S+) and no cell fusion when treated with K-874A VHH (S+K-874A+). Inset: magnified view of dotted square shown in left panels. (**G**) ELISA results show K-874A (left) and ACE2 (right) bound to immobilized recombinant S protein in a dose-dependent manner. Immobilized S protein was detected by serially diluted K-874A (1 to 0.002 μg/well) and horseradish peroxidase (HRP)-conjugated anti-VHH antibody (left), or by serially diluted ACE2 protein (20 to 0.0625 μg/ml) and anti-ACE2 antibody and HRP-conjugated secondary antibody (right). Binding of ACE2 to S protein was unaffected by K-874A (1 μg/well) treatment.

With its strong binding affinity and specificity to SARS-CoV-2, we investigated K-874A as a potential therapeutic drug for COVID-19. We infected African green monkey kidney (VeroE6) cells expressing transmembrane serine protease 2 (VeroE6/TMPRSS2) with SARS-CoV-2 (KUH003) and determined how well K-874A inhibited SARS-CoV-2 infection in the cells by quantitative real-time polymerase chain reaction (qRT-PCR). The half-maximal inhibitory concentration (IC_50_) calculated from RNA copies was 5.74±2.6 μg/mL (Fig 2A).

To determine whether K-874A VHH neutralizes SARS-CoV-2 by preventing the virus from attaching to the host cell, we compared the early phases of infection in cells infected with K-874A-treated virus and untreated virus. VeroE6/TMPRSS2 cells were incubated with K-874A-treated SARS-CoV-2 and untreated virus for 1 hr, and the number of RNA copies that were attached or incorporated into the cells were determined by qRT-PCR. K-874A treatment did not change the levels of virus attachment or early incorporation in the cells (Fig 2B). At 6 hr post-infection, the number of viral RNA copies in cells infected with untreated virus were increased more than 400 times, but the numbers were remarkably inhibited in cells infected with K-874A treated virus (Fig 2C). Additionally, expression of the viral protein, NSP8, and double-stranded RNA (dsRNA), which is generated during viral RNA replication, were suppressed in cells infected with K-874A-treated viruses (Fig 2D). These results indicated that K-874A did not reduce viral attachment.

We further investigated whether K-874A acts by inhibiting the virus from fusing with the host cell. VeroE6/TMPRSS2 cells express the ACE2 receptor and the TMPRSS2 protease on its surface. To study viral fusion, we transduced the VeroE6/TMPRSS2 cells with a lentivirus coding for the viral S protein into the cells. When the S protein from one VeroE6/TMPRSS2 cell binds to the ACE2 receptor on an adjacent VeroE6/TMPRSS2 cell, the TMPRSS2 protease cleaves and activates the S protein for fusion. To quantify this cell fusion, we used HiBiT technology, which involves the binding of HiBiT (a small 11–amino acid peptide) with a larger subunit, called LgBiT, to form a complex with luciferase activity. VeroE6/TMPRSS2 cells expressing HiBiT or LgBiT were produced and co-cultivated. The HiBiT-LgBiT complex and the resulting luciferase activity form only when the S protein from one cell binds to the ACE2 receptor on an adjacent cell and their membranes are fused. We found that treating VeroE6/TMPRSS2 cells with K-874A suppressed cell fusion in a dose-dependent manner (Fig 2E). Cells without S protein (S ˗) did not fuse, whereas those expressing S protein but not treated with K-874A (S+) did fuse. Optical micrographs confirmed these results (Fig 2F). Direct binding of K-874A or recombinant ACE2 to immobilized recombinant S protein in ELISA further showed that both K-874A (left panel in Fig 2G) and ACE2 (blue line on right panel in Fig 2G) bound to S protein in a dose-dependent manner. However, K-874A did not block ACE2-S protein interaction (red line on right panel in Fig 2G). These findings suggest that K-874A does not prevent the virus from attaching to the ACE2 receptor on the cell surface. Rather, K-874A prevents the virus from entering the cell by blocking the viral membrane from fusing with the host cell.

**Fig 3:**
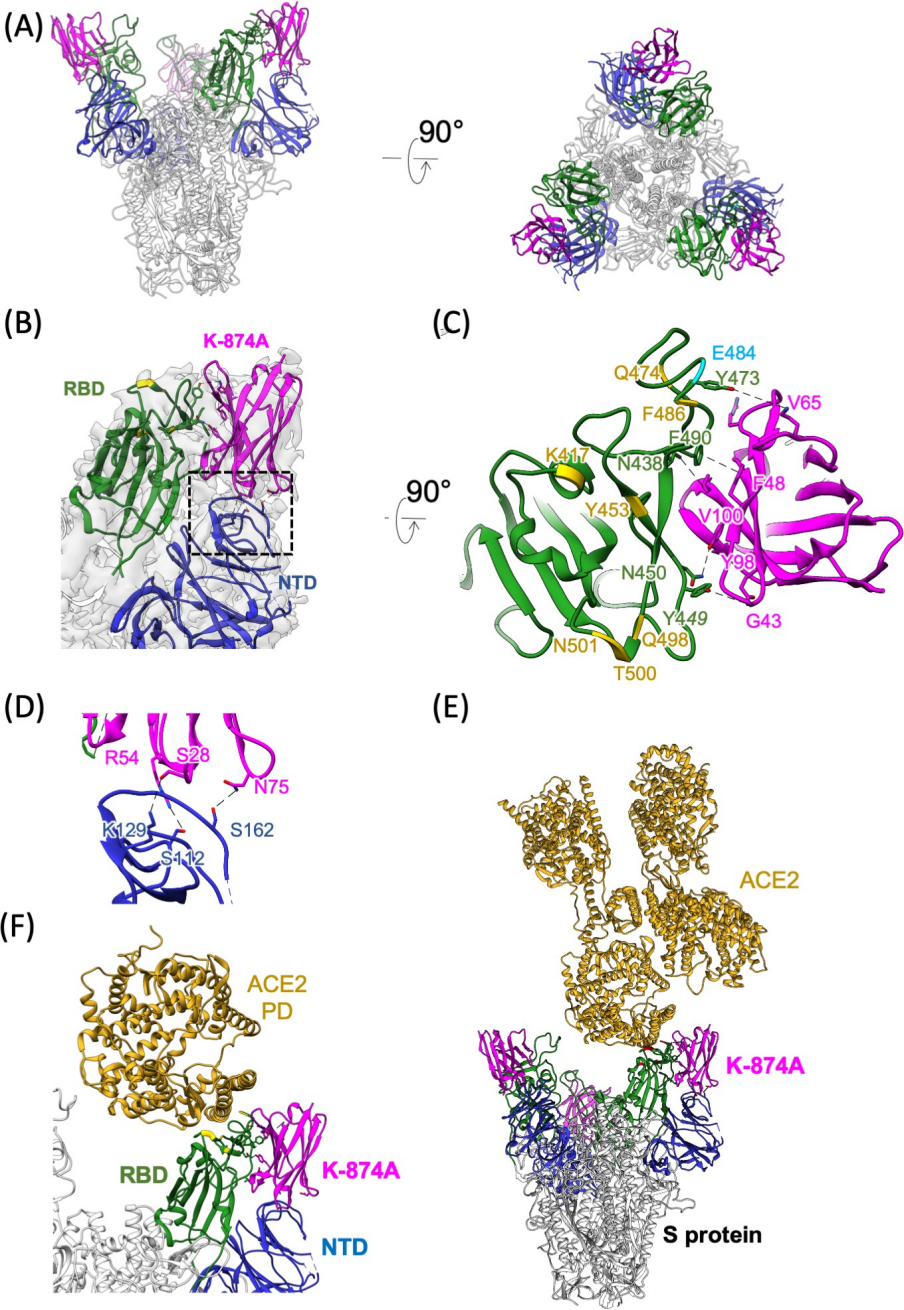
K-874A-binding region on the S protein in the major cryo-EM map (type 1). (**A**) A ribbon model of S protein trimer and K-874A VHHs. The coloring follows the standard designation of K-874A VHH (magenta), NTD (blue) and RBD (green). (**B**) An enlarged view of RBD, NTD and K-874A in (A). (**C**) A view of the structure in (B) rotated 90°. Residues that interact with ACE2 are shown in yellow. N501 and E484 (cyan) are amino acid residues frequently mutated. Possible chemical interactions between RBD and K-874A are shown with black dotted lines. (**D**) Possible chemical interactions between K-874A and NTD shown with black dotted lines. The viewing area was indicated by the dotted square in (B). (**E**) The binding of K-874A and ACE2 on RBD. (**F**) A close-up view of the interactions between the peptidase domain (PD) of ACE2, K-874A, RBD, and NTD. K-874A does not interfere the binding of ACE2.

To estimate the K-874A binding region on the S protein, we reconstructed the cryo-electron microscopy (cryo-EM) structures of the recombinant S protein trimer (S1 Fig) and its complex with K-874A complex (S2 Fig), and fitted each molecular model to the maps (S3G–S3I Fig). As a result, we acquired two types of the complex structures, named type 1 and type 2 (S2 Fig). The ratio of type 1 and type 2 particles was approximately 9:1. The type 1 structure showed that K-874A was located in the vacant space between the NTD and RBD of the S protein (Figs 3A–3D, and S3B, S3E and S3H). In the prefusion state, the RBD moves upwards to bind ACE2 [17,18]. However, the RBD in the type 1 structure did not rise up after binding K-874A and moved laterally to the outside of the S protein trimer (S5A Fig). The NTD moved down after binding of K-874A. Amino acid residues in CDR1, CDR2, and N-terminal of K-874A formed polar bonds with the NTD of the S protein, whereas amino acid residues of CDR2 and CDR3 bonded hydrophobically with the RBD (Fig 3B–3D). In the type 2 structure, K-874A was bound only to the RBD and not to the NTD (S3C, S3F, S3I and S4 Figs). The S protein trimer was in the prefusion state with RBD moving upwards (S5B Fig). All amino acid residues that interact with ACE2 were placed in the upward state of RBD, even when the RBD was bound to K-874A, demonstrating that ACE2 binds to RBD even in the bound state of K-874A (Fig 3E and 3F). These findings demonstrate that K-874A neutralizes SARS-CoV-2 via a different route that did not involve ACE2 binding.

### K-874A VHH neutralizes B.1.1.7 variant but no other variants

We evaluated the binding affinity and the neutralization effect of K-874A against variants prevalent to date. Calu3 cells were infected with each variant and incubated for an hour. After removing the inoculum and washing, Calu3 cells were incubated with fresh new medium, including K-874A for 2 days. The numbers of RNA copies of Wuhan, KUH003 (D614G), and B.1.1.7 (Alpha) in the medium at day 2 were slightly reduced (S8A Fig), suggesting that newly produced progeny viruses were captured by K-874A and could not infect the cells again. Numbers of RNA copies of B.1.351 (Beta), P.1 (Gamma), and B.1.617.2 (Delta) did not change with or without K-874A. To further investigate the infectivity of each progeny in the culture supernatant, the number of RNA copies was adjusted to 10,000, and the TCID_50_ was calculated after infecting Vero/TMPRSS2 cells. Infectivity of Wuhan, KUH003 (D614G), and B.1.1.7 (Alpha) were remarkably depressed by K-874A treatment, and the infectivities of B.1.351 (Beta), P.1 (Gamma) and B.1.617.2 (Delta) were not (S8B Fig). Furthermore, the binding affinity of K-874A to S protein from each variant was evaluated by ELISA. K-874A bound to Wuhan- or B.1.1.7 (Alpha)-derived-S, but not to B.1.351 (Beta), P.1 (Gamma), B.1.429 (Epsilon), B.1.525 (Eta) and B.1.617 (Kappa) (S8C Fig). Neutralized variants did not have the mutation in K-874A binding motif at amino acids 450–490 of the S protein; however, un-neutralized variants had some mutations. These finding suggested that it would be neutralized if K-874A is strongly bound to S protein and targeting to applicable motif of S proteins may lead to a highly effective VHH to neutralize these un-neutralized variants.

### K-874A VHH prevents viral replication in lung organoids

**Fig 4:**
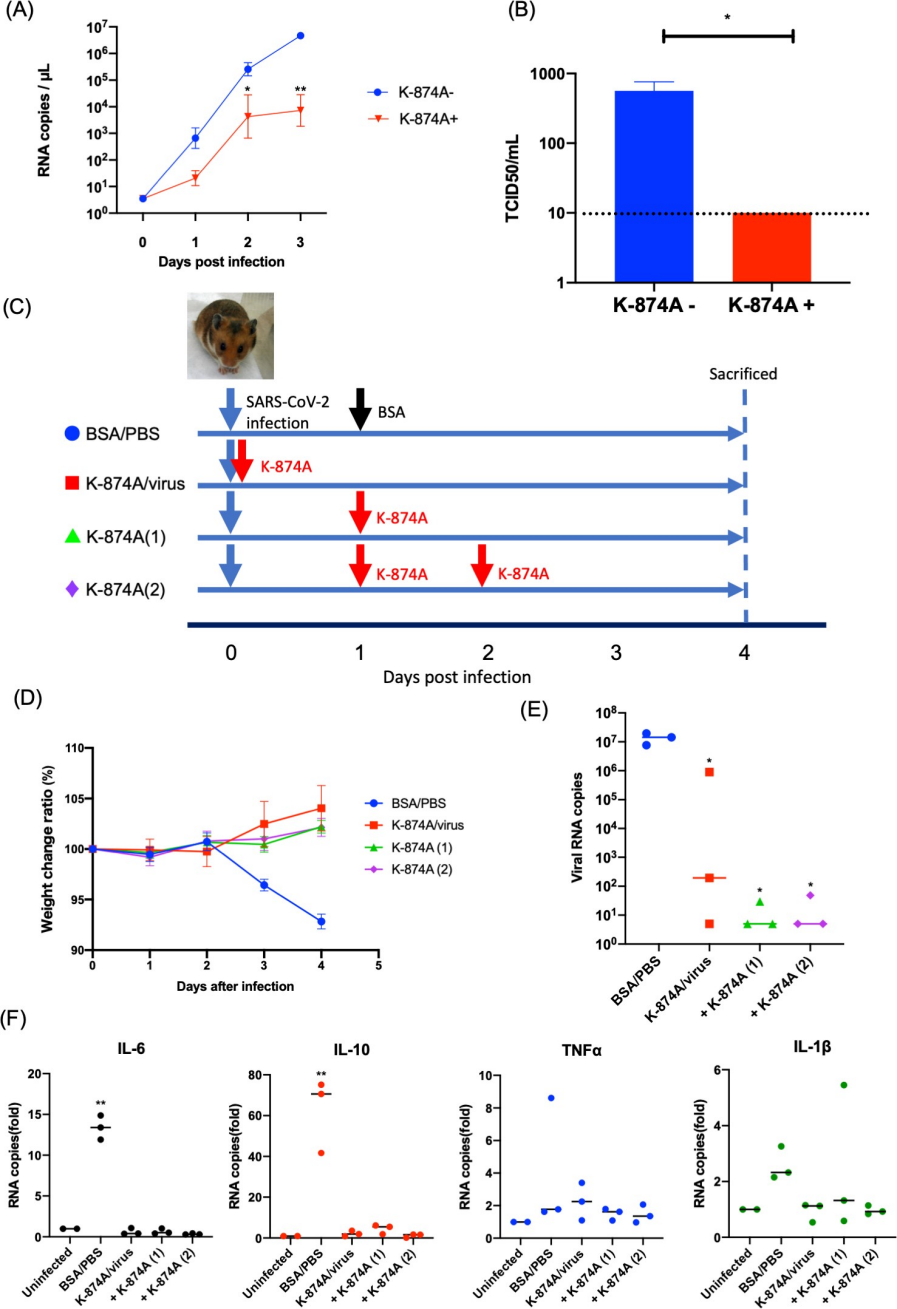
K-874A VHH treatment inhibits SARS-CoV-2 replication in an alveolar organoid model and improves viral symptoms in Syrian hamsters. (**A**) Graph shows infected alveolar organoid treated with K-874A-containing medium for 3 days released fewer RNA copies (1x10^4^ RNA copies/μL) into the culture supernatant than organoids that were not treated with K-874A (>1x10^5^ RNA copies/μL). (N = 5, * P<0.05, ** P<0.005, Welch’s t-test). Data are from a representative experiment of two independent experiments. (**B**) Virus progeny in each supernatant at Day 3 were adjusted to 1x10^4^ RNA copies and the infectivity was compared between K-874A treated and untreated. The infectivity of K-874A untreated was 565±195 TCID_50_/ml, and K-874A-treated was lower than 10 TCID_50_/ml. Virus infectivity was determined in VeroE6/TMPRSS2 cells. Error bars indicate mean ± SEM. Black dotted line indicates detection limit. (N = 5, * P<0.05, Welch’s t-test) (**C**) Time schedule for inoculation and K-874A administration to Syrian hamsters. All animals were infected with 2x10^3^ TCID_50_ of SARS-CoV-2. Untreated animals were treated with BSA/PBS (blue circle) on Day 1 and Day 2 post-infection. K-874A-treated groups were treated with either a single dose immediately before infection (K-874A/virus; red square) or 1 day after infection (+ K-874A (1); green triangle), or two doses at Day 1 and Day 2 after infection (+ K-874A (2); purple rhombus). Each dose was 3 mg K-874A per animal. (N = 3 for infected with untreated or treated) (**D**) Unlike BSA/PBS-treated animals, all K-874A-treated animals showed no weight loss. Weight at Day 0 is 100%. (**E**) Amounts of viral RNA in the lung homogenates at Day 4, as determined by qRT-PCR, show all K-874A-treated animals had fewer viral RNA copies in their lungs than untreated animals. (N = 3 for infected and N = 2 for uninfected, *P<0.05, Dunnett’s multiple comparison test) (**F**) qRT-PCR measurements of lung homogenates collected on Day 4 show untreated animals expressed elevated levels of inflammatory cytokines IL-6 and IL-10 but not TNF-α and IL-1β. Cytokine levels in K-874A-treated animals were similar to uninfected animals. (N = 3 for infected and N = 2 for uninfected, **P<0.01, Dunnett’s multiple comparison test).

Because SARS-CoV-2 targets lung tissues and induces severe respiratory disease, we evaluated K-874A VHH in a human lung–derived alveolar organoid that is susceptible to SARS-CoV-2 and releases high levels of progeny virus into the culture supernatant [19,20]. To determine if K-874A blocks production of virus progeny in infected alveolar organoids, we incubated the organoid cells with SARS-CoV-2 for 1 hr before treating the infected cells with K-874A-containing medium for 3 days. Untreated cells released >1x10^7^ RNA copies/μL into the culture supernatant at day 3, and VHH treatment reduced the progeny production to 1x10^4^ RNA copies/μL (Fig 4A), indicating that K-874A reduced the production of virus progeny. To test the infectivity of virus progeny produced from K-874A-treated and untreated infected cells, we adjusted each viral progeny to 1x10^4^ RNA copies and incubated with fresh VeroE6/TMPRSS2 cells. Untreated virus progeny showed 565 TCID_50_/ml, and K-874A-treated virus progeny showed fewer than 10 TCID_50_/ml (Fig 4B). These data suggested that the K-874A-treated progeny have a lower infectivity. This is likely due to K-874A VHH binding to the S protein of the virus progeny, preventing them from infecting other cells.

### Infected Syrian hamsters recover after K-874AVHH treatment

We further tested K-874A VHH as a potential therapeutic drug for COVID-19 in the Syrian hamster model. When infected with SARS-CoV-2, Syrian hamsters lose weight but spontaneously heal by 14 days post-infection [21]. To determine if K-874A improves the symptoms of COVID-19 infection, we infected 6-week-old male Syrian hamsters with SARS-CoV-2 (2 x 10^3^ TCID_50_) and administrated different regimens of K-874A intranasally. The animals were treated with a single dose of K-874A just before the infection or at 1 day after the infection, or multiple doses at 1 and 2 days after the infection (Fig 4C). Each dose of K-874A was 30 mg/kg, which was determined to be the maximum dose that can be administrated. All infected animals treated with K-874A did not lose weight, but infected control animals treated with bovine serum albumin in phosphate-buffered saline (BSA/PBS) lost weight 3 days after the infection (Fig 4D). Further, all K-874A-treated hamsters, regardless of treatment regimen, had fewer viral RNA copies in their lung tissues than BSA/PBS-treated hamsters 4 days after the infection (Fig 4E). Because cytokines are upregulated after an infection [22,23], we compared the mRNA levels of several cytokines in the infected lung tissues of the untreated and K-874A-treated animals. Untreated animals showed elevated levels of IL-6 and IL-10 but not IL-1β and TNF-α, in their lungs 4 days after the infection. All K-874A-treated animals, regardless of the treatment regimen, displayed cytokine levels that were similar to uninfected animals (Fig 4F). When different doses (1.2, 6, or 30 mg/kg) of K-874A were administered in a two-dose regimen (on Days 1 and 2 post-infection) (S9A Fig), animals receiving the lowest K-874A dose displayed the highest weight loss and viral RNA copies in their lung tissues (S9B and S9C Fig). Their IL-6 and IL-10, but not IL-1β and TNF-α, levels were also higher than those receiving higher doses of K-874A (S9D Fig). These data demonstrate that K-874A improved the symptoms of COVID-19 infection and in a dose-dependent manner.

## Discussion

Besides vaccines, therapeutic drugs are urgently needed to treat COVID-19 infections. VHH are promising drug candidates because they are more stable and cheaper to produce than human monoclonal antibodies. They are also amenable to nasal administration, allowing high concentrations of drugs to reach directly to infected lungs and remain effective for longer. Indeed, nasal administered VHH against RSV is effective for 3 days [6]. However, due to their low molecular weight, VHH monomers have very short half-lives in the blood stream [4]. VHHs under development as antivirals need to be multivalent or modified with human antibody fragments to enhance their antiviral effect or extend their half-life. A previous report showed VHH that bound to the S protein of SARS-CoV-2 could neutralize the virus only when VHH was fused with an Fc domain of a human antibody [7].

Here, using *in vitro* selection, we identified a standalone anti-SARS-CoV-2 S1 VHH that binds to the S protein of SARS-CoV-2 with a higher or equivalent affinity or neutralizing ability than previous VHHs or single domain antibody [7–12]. Furthermore, it displays excellent neutralizing ability in VeroE6/TMPRSS2 cells and human normal alveolar-derived cells. Although accurate comparison the affinity or neutralizing IC_50_ between labs is difficult, the binding affinity of K-874A was 1.4 nM and IC_50_ was 5.74±2.6 μg/ml in contrast to reported VHHs (e.g., VHH-72 (K_D_ = 38.6 nM, IC_50_ = 0.2 μg/ml) [7], n3088 (K_D_ = 3.7 nM, IC_50_ = 3.3 μg/ml) [8], Ty1 (K_D_ = 5–10 nM, IC_50_ = 0.77 μg/ml) [9], Sb23 (K_D_ = 10 nM, IC_50_ = 0.6 μg/ml) [10], H11-H4 (K_D_ = 5 nM, IC_50_ = 61 nM) [11], Nb11-59 (K_D_ = 20 nM, IC_50_ = <1 μg/ml) [12]). Fc-fusion was necessary to achieve the IC_50_ of these previous reported VHHs, except n3088 and Nb11-59, and these mainly targeted ACE2 binding.

We found that this VHH neutralizes SARS-CoV-2 by preventing the virus membrane from fusing with the host cell membrane. Cryo-EM analysis of the S protein-VHH complex revealed that the VHH binds between the RBD and NTD region on the S protein, rather than at the interface of the RBD and ACE2. Neutralization of SARS-CoV-2 by NTD-binding human antibody was also reported [24]; however, it was clearly different domain of previous reported VHHs (S10 Fig). Our cryo-EM data indicated that K-874A binding induced a conformational change of S protein (S5 Fig), but more investigation is required to determine the neutralizing mechanism independent of RBD-ACE2 binding inhibition. In human lung-derived alveolar organoids, the VHH reduced the production of virus progeny. Moreover, virus progeny produced from VHH-treated infected cells had a lower infectivity than those from untreated infected cells, suggesting that the VHH prevents the virus from spreading to uninfected cells or persons. Intranasal administration of our VHH to SARS-CoV-2-infected Syrian hamsters prevented weight loss, viral replication in the lungs and upregulation of cytokines typically caused by SARS-CoV-2 infection. K-874A did not bind to S protein derived from variants that cannot be neutralized, indicating that VHHs targeting the RBD-NTD region might effectively neutralize these variants. Furthermore, combinations of VHHs targeting different epitope (e.g., the NTD-RBD targeted VHH with RBD-targeting VHH) may lead to development of cocktail therapeutics. Since new variants will likely appear in the future, we need to develop neutralizing VHHs targeting the conserved region.

Our VHH has several advantages. Inhaling a human monoclonal antibody against SARS-CoV-2 can inhibit virus replication in the lung and nasal turbinate [25]. This suggests that the therapeutic benefits of our VHH could also be delivered using a nebulizer. Nasal administration via a nebulizer is expected to lower the amount of VHH entering the blood stream, which could reduce the risk of immunoreaction against VHH on repeated use. In support of the use VHHs for therapeutics to patients, nebulization and humanization of VHH were successfully implemented without impairing the stability or the neutralizing ability [12]. Further, because our VHH displays striking antiviral effects without the additional Fc domain, its risk to Fc-related antibody-dependent enhancement is likely to be low.

Abnormal secretion of cytokines is a hallmark of serious cases of COVID-19 infections [26]. While it remains unknown why such cytokine storms occur, ACE2 may be an interferon-stimulated gene that SARS-CoV-2 could exploit to enhance infection [3]. Because our VHH prevents viral fusion, we believe that it can reduce viral infection and, in turn, inhibit interferon upregulation. The efficacy of our VHH could also be improved if used in conjunction with antibodies that block ACE2-RBD binding.

## Materials and methods

### Ethics statement

Animal experiments were approved by the President of Kitasato University through the Institutional Animal Care and Use Committee of Kitasato University (20–031), and performed in accordance with the Guidelines for Animal Experiments of Kitasato University. Human normal lung organoid experiments for SARS-CoV-2 infection were approved by the ethics committees of Keio University School of Medicine and Kitasato Institute Hospital.

### Preparation of cDNA display library

A cDNA display library (200 pmol-scale) used for the 1^st^ round of selection (R1) was synthesized from an initial VHH-encoding DNA library (predicted diversity, 1x10^13^), provided by Epsilon Molecular Engineering Co. Ltd. (Japan). A cDNA display library (6-pmol scale) used at the 2^nd^ and 3^rd^ rounds of selection (R2 and R3, respectively) was synthesized from the selected DNA library of each previous round. Translation of the DNA library was performed using T7 RiboMAX Express Large-Scale RNA Production System (Promega, USA), according to the manufacturer’s instructions. The DNase-treated mRNA mixture was purified using Agencourt RNAClean XP beads (Beckman Coulter Genomics). The purified mRNA was hybridized to cnvK (riboG) linker (Epsilon Molecular Engineering) at the 3′-terminal region in 50 mM Tris-HCl (pH 7.5) with 200 mM NaCl under the following annealing conditions: heating at 90°C for 2 min, followed by lowering the temperature to 70°C at a rate of 0.1°C/s, incubating for 1 min, then cooling to 25°C at a rate of 0.1°C/s, incubating for 30 sec, and then stored at 4°C until use. The mixture was irradiated with UV light at 365 nm using a handheld UV lamp (6W, UVGL-58, 254/365 nm, 100V; Analytik Jena) for 5 min to obtain mRNA-linker complex.

The mRNA-linker complex was translated using a Rabbit Reticulocyte Lysate System, Nuclease-Treated (Promega) at 37°C for 15 min. To synthesized mRNA-linker-VHH complex, KCl and MgCl_2_ were added to final concentrations of 900 and 75 mM, respectively, and the mixture was incubated at 37°C for 20 min.

EDTA (final concentration: 70 mM) and an equal volume of 20 mM Tris-HCl buffer (pH 8) containing 2 mM EDTA, 2 M NaCl and 0.2% Tween 20 were added, and the mRNA-linker-VHH complex was immobilized in 60 μL of Dynabeads MyOne streptavidin C1 magnetic beads (Thermo Fisher Scientific) at 25°C for 30 min. The beads were washed two times with 60 μL of wash buffer (10 mM Tris-HCl (pH 8), 1 mM EDTA, 1 M NaCl and 0.1% Tween 20). The immobilized library was then reverse-transcribed by ReverTra Ace (TOYOBO) at 42°C for 30 min. The beads were washed two times with 200 μL of wash buffer (20 mM sodium phosphate buffer, 0.5 M NaCl, 5 mM imidazole, and 0.05% Tween 20 (pH 7.4)) and then, 30 μL of RNase T1 (Thermo Fisher Scientific) prepared with the wash buffer was added to the beads. The mixture was incubated for 15 min 37°C to release the cDNA-linker-VHH complex from the beads. The supernatant containing cDNA-linker-VHH complex was purified using His Mag Sepharose Ni beads (GE Healthcare). The supernatant was added to 20 μL of the beads and incubated at 25°C for 30 min. The beads were washed with 200 μL of wash buffer (20 mM sodium phosphate buffer, 0.5 M NaCl and 20 mM imidazole (pH 7.4)), and then incubated in 10 μL of elution buffer (20 mM sodium phosphate buffer, 0.5 M NaCl and 250 mM imidazole, 0.05% Tween 20 (pH 7.4)) at 25°C for 15 min. The elution collected was stored at 16°C until use.

### *In vitro* selection of VHHs using cDNA display

The C-terminal polyhistidine tagged-SARS-CoV-2 S1 subunits (Val16-Arg685) (YP_009724390.1) (#40591-V08H, Sino Biological) were used as target molecules for screening. Microtiter wells of Nunc-Immuno Plate II (Thermo Fisher Scientific) were coated overnight at 4°C with 100 μL of 100 (R1), 10 (R2), or 1 μg/mL (R3) recombinant SARS-CoV-2 S1 subunits-His tag that was prepared with PBS. The S1 subunits-coated wells were blocked with 200 μL of 3% BSA in PBST for 2 hr at room temperature, and then washed at three times with 200 μL of HBST. 100 μL of VHH-cDNA complex library was prepared as follows: 50 μL of HEPES buffer containing 1% BSA was added to 50 μL of VHH-cDNA conjugate library for R1, 50 μL of HBST containing 1% BSA, 25 μL of HBT was added to 50 μL of cDNA display library for R2 and R3, respectively. The VHH-cDNA complex library was added to the target-fixed well, incubated for 1 hr at room temperature. The residual VHH-cDNA conjugates were removed by washing at 10 times with 200 μL of HBST. The target-binding VHH-cDNA complex was eluted by adding 100 μL of 100 mM Tris (pH 11), gently pipetting, and incubating for 10 min at 37°C. The elution was immediately transferred to PCR cocktails prepared with KAPA HiFi HotStart ReadyMix (2x) (Kapa Biosystems, USA), forward primer (5′-GATCCCGCGAAATTAATACGACTCACTATAGGGGAAGTATTTTTACAACAATTACCAACA-3′), and reverse primer (5′-TTTCCACGCCGCCCCCCGTCCT-3′). The PCR cycle conditions consisted of 95°C for 2 min, followed by 22 cycles (R1) or 24 cycles (R2 and R3) of 98°C for 20 sec, 68°C for 15 sec, and 72°C for 20 sec, and then 72°C for 5 min. The PCR products were purified using Agencourt AMPure XP beads (Beckman Coulter Genomics), according to the manufacturer’s instruction. The purified DNA libraries were used for translation, as described above, to prepare cDNA display libraries used at the next selection round.

### Sequencing of the DNA library

DNA libraries obtained at R2 and R3 were used as templates in amplicon PCR for Illumina sequencing. The DNA library was quantified using PicoGreen dsDNA reagent kit (Thermo Fisher Scientific) and prepared at 100 ng/mL with nuclease-free water. 1^st^ PCR was performed with 12.5 μL of KAPA HiFi HotStart ReadyMix (2x) (Kapa Biosystems), 0.5 μL of 10 μM forward primer (5′-TCGTCGGCAGCGTCAGATGTGTATAAGAGACAGNNNNATGGCTGAGGTGCAGCTCGTG-3′), 0.5 μL of 10 μM reverse primer (5′-GTCTCGTGGGCTCGGAGATGTGTATAAGAGACAGNNNNTGATGATGATGGCTACCACCTCCCG-3′), 9 μL of nuclease-free water, and 2.5 μL of template DNA. The PCR cycle conditions consisted of 95°C for 3 min, followed by 16 cycles of 98°C for 20 sec, 62°C for 15 sec, and 72°C for 20 sec, and then 72°C for 5 min. PCR clean-up was performed with Agencourt AMPure XP beads (Beckman Coulter Genomics). Subsequently, index PCR was performed with 12.5 μL of KAPA HiFi HotStart ReadyMix (2x) (Kapa Biosystems), each 1 μL of 10 μM forward and reverse primers from Nextera XT Index Kit v2 (Illumina), 8 μL of nuclease-free water, and 2.5 μL of template DNA. The PCR cycle conditions consisted of 95°C for 3 min, followed by 8 cycles of 98°C for 20 sec, 55°C for 15 sec, and 72°C for 30 sec, and then 72°C for 5 min. After magnetic bead-based purification of PCR products, the library concentration was measured with PicoGreen dsDNA reagent kit (ThermoFisher Scientific) and prepared to 4 nM with nuclease-free water. The library was diluted to a final concentration of 7 pM and 5% of PhiX DNA (Illumina) was added. Sequencing was performed with the Miseq Reagent Nano kit V2 500 cycle using a MiSeq 2000 (Illumina), according to the manufacturer’s instructions.

### Data analysis

Dell Mobile Precision 7520 (Xeon E3-1535M v6 (Quad core, 3.1 GHz, 4.2 GHz turbo), 1TB SSD, memory: 64 GB (4x16 GB), 2,400 MHz DDR4 ECC SDRAM) was used to analyze sequencing data. The raw Illumina paired-end reads that passed through the Q30 filter were merged using PEAR software [27]. with default parameter on Linux. Nucleotide sequences encoding VHHs were extracted using combination of basic Linux command lines specifying typical nucleotide sequences conserved on VHHs frame regions. The VHHs-encoding sequences were translated based on standard genetic code using MEGA X software [28].

### Purification of VHHs

*Bacillus subtilis* 168, which is deficient for nine proteases (i.e., *epr*, *wprA*, *mpr*, *nprB*, *bpr*, *nprE*, *vpr*, *aprE* and *aprX*) [29] and contains a sigma factor for a sporulation (*sigF*)-deficient mutant was used (JP4336082B2). *B*. *subtilis* was precultured in L medium (1% tryptone, 0.5% yeast extract, and 0.5% NaCl) and cultured in 2xL-Mal medium (2% tryptone, 1% yeast extract, 1% NaCl, 7.5% maltose hydrate, and 7.5 μg/mL MnSO_4_) with 15 μg/mL tetracycline at 30°C 72 hr.

Supernatants were subjected to SDS-PAGE and western blotting. SDS-PAGE used SuperSep Ace 15–20% Tricine Gel (Wako), and then proteins in the gel were transferred to PVDF membrane using Trans-Blot Turbo Mini PVDF Transfer Packs (Bio-Rad) and Trans-Blot Turbo System (Bio-Rad). PVDF membranes were treated with 6xHis Tag Monoclonal Antibody (3D5) HRP (Thermo Fisher Scientific) for 6xHis-tagged proteins, and ANTI-FLAG M2-peroxidase (HRP)-conjugated (Sigma Aldrich) for FLAG (DYKDDDDK)-tagged proteins in iBind Western System (Thermo Fisher Scientific). Each tagged protein was visualized by 1-Step Ultra TMB-Blotting Solution (Thermo Fisher Scientific).

His-tagged K-874A was purified from the supernatants of culture medium using Ni-NTA agarose beads (Wako) and resuspended into PBS with 30 mM Imidazol. The K-874A-FLAG fraction was collected from the supernatants using an Amicon Ultra Centrifugal Filter Unit (Merck) and resuspended into PBS with 30 mM imidazol. The purified VHHs were stored at 4°C until use.

### ELISA for evaluation of the binding specificity of K-874A

Microtiter wells of Nunc-Immuno Plate II (Thermo Fisher Scientific) were coated overnight at 4°C with 100 μL of His-tagged recombinant human-CoV-HKU1 and OC43, SARS-CoV-2 (Sino Biological) and SARS-CoV S1 subunits (The Native Antigen Company) diluted in PBS. The S1 subunits-coated wells were blocked with 200 μL of 5% skim milk in PBS for 1 hr at room temperature, and then washed at three times with 200 μL of PBST. 100 μL of 20 μg/mL K-874A-FLAG prepared with PBST were added to the wells, incubated for 1 hr, and then washed at three times with 200 μL of PBST. The binding of K-874A-FLAG to S1 subunits-His tag was detected by incubating for 1 hr at room temperature with mouse monoclonal ANTI-FLAG M2-Peroxidase (Merck) diluted with PBST. The wells were washed three times with PBST, and then 100 μL of substrate buffer prepared with an o-phenylenediamine dihydrochloride substrate tablet (Thermo Fisher Scientific), 10x Stable Peroxide Substrate Buffer (Thermo Fisher Scientific), and water. After incubating for 30 min at room temperature, the absorbance at 450 nm was immediately measured with Microplate Reader Infinite M1000 PRO (TECAN).

### Biolayer interferometry

Binding affinities of K-874A and S1 protein were determined using biolayer interferometry (BLI) of Octet RED 384 (Pall Life Sciences, USA). The kinetic buffer was PBST for BLI. The temperature was fixed at 25°C. 50 μl of each measurement solution was added to a 384-well black plate (Fortebio), and the measurements were performed as below. 1) The loading baseline was measured in kinetic buffer for 30 sec. 2) K-874A-6xHis was immobilized on a HIS1K biosensor (Fortebio) and loaded until a 0.3-nm signal was achieved. 3) The measurement baseline was measured in kinetic buffer for 30 sec. 3) The loaded sensors were dipped into twofold serial dilutions from 245.8 nM of the SARS-CoV-2 (2019-nCoV) Spike S1-Fc Recombinant Protein (Sino Biological) to measure a 180-sec specific binding at the association step. 4) The dissociation was obtained by dipping the biosensors once more time into the kinetic buffer for 240 sec. The data were analyzed using Fortebio Octet analysis software (Date Analysis HT Version 11.1.2.48) (Fortebio) and kinetic parameters were determined using a 1:1 monovalent binding model.

### Cells and viruses

VeroE6/TMPRSS2 were purchased from JCRB Cell Bank and preserved in our laboratory. Calu3 was gently gifted from Dr. Matsuyama in NIID. SARS-CoV-2, 2019-nCoV JPN/TY/WK-521, B1.1.7 (Alpha), B.1.351(Beta), P.1 (Gamma) and B.1.617.2 (Delta) were provided from National Institute of Infectious Diseases (NIID, Japan). KUH003 (Accession number LC630936) [20] is isolated from nasal swab of COVID-19 patients in Tokyo. KUH003 has D614G.

### Neutralization assay

2.5 x10^3^ TCID_50_ of SARS-CoV-2 (Multiplicity of Infection: MOI = 0.05) was incubated with serial diluted VHHs for 2 hours at 37°C and subsequently for 24 hr at 4°C. VHH-treated virus was incubated with 5.0x10^4^ of VeroE6/TMPRSS2 cells for 1 hr at 37°C, and the wells were washed to remove unbound viruses. At 24 hr after infection, the culture supernatant was used to determine the number of RNA copies of virus by qRT-PCR (SARS-CoV-2 Detection Kit, TOYOBO), according to manufacturer’s protocol. IC_50_ was determined 1 day after infection.

### Neutralization assay using Calu3 cells

2.5 x10^3^ TCID_50_ of each SARS-CoV-2 variant (MOI = 0.05) was incubated with 5.0 x10^4^ of Calu3 cells for 1 hr at 37°C, and the wells were washed to remove unbound viruses. After washing, the cells were incubated with or without 150 μg/ml of K-874A for 2 days. At 2 days after infection, the culture supernatant was used to determine the number of RNA copies of virus by qRT-PCR (SARS-CoV-2 Detection Kit, TOYOBO), according to manufacturer’s protocol. Based on the RNA copies, each variant in the culture supernatant was adjusted 10,000 RNA copies and was determined their TCID_50_ by infecting to VeroE6/TMPRSS2 cells.

### ELISA for evaluation of the binding specificity of K-874A to variants

20 μg/ml of K-874A was immobilized on Pierce Nickle Coated Plates, Clear, 96-well (Thermo Fisher Scientific). PBST was used for washing after each step. K-874A-coated wells were blocked with 5% skim milk in PBST for 1 hr. Recombinant S proteins produced by HEK293 cells were purchased from BioServ. Each S protein was diluted 5, 50, or 500 ng/ml and 100 μl of diluted S protein was incubated for 1 hr. The S protein captured by K-874A was detected with anti-rhodopsin [1D4] (Abcam) mouse monoclonal antibody and anti-mouse IgG goat polyclonal antibody-HRP (Abcam). The o-phenylenediamine dihydrochloride substrate tablet (Thermo Fisher Scientific) dissolved with Stable Peroxide Substrate Buffer (Thermo Fisher Scientific) was used as a substrate. The absorbance at 450 nm was measured with GloMax Explorer System (Promega).

### SARS-CoV-2 binding on and early replication in Vero/TMPRSS2 cells

2.5 x10^4^ TCID_50_ (MOI = 0.5) of SARS-CoV-2 was incubated with 150 μg/ml of VHHs for 2 hr at 37°C and then 24 hr at 4°C and inoculated to 5.0 x10^4^ VeroE6/TMPRSS2 cells for an hr. Similarly, to compare early RNA replication in the infected cells, infected cells were collected at 3 or 6 hr after infection. Total RNA was extracted from the collected cells with Nucleospin RNA (TAKARA). Numbers of RNA copies of the virus were determined by qRT-PCR (SARS-CoV-2 Detection Kit, TOYOBO), according to manufacturer’s protocol.

### Cell fusion assay

To quantify the cell fusion induced by S protein, HiBiT technology (Promega) was applied. HiBiT was linked to the C-terminal of ZsGreen (TAKARA) and transduced by lentivirus vector LVSIN-IRES-puro, and LgBiT was transduced by lentivirus vector LVSIN-IRES-Hyg [30]. Equal numbers of ZsGreen-HiBiT-expressing cells and LgBiT-expressing cells were plated on a 96-well plate (1603101, Thermo Fisher Scientific). S protein was transduced by lentivirus vector. At 14 hr after transduction, the culture supernatant was replaced to Opti-MEM (ThermoFisher Scientific), and 25 μL of diluted Nano-Glo live solution (Promega) was added into the each well. After mixing, the relative luminescence was measured by Ensight (Perkin Elmer).

### ELISA assay for direct interaction of recombinant ACE2 and S protein

Diluted recombinant S protein (2 μg/mL) (#RP012383LQ, ABclonal) with PBS was incubated to immobilize on the maxisorp plate (464718, Thermo). After blocking with 1% BSA/PBS, 1 μg of K-874A diluted in 1%BSA/PBS was incubated to bind to S protein. Then, serial diluted recombinant ACE2 (from 1 to 0.06 μg/ml) (ab151852, abcam), subsequent rabbit anti-ACE2 antibody (HPA000288, Atlas Antibodies) and HRP-conjugated anti-rabbit IgG (7074S, Cell signaling) were incubated. To detect K-874A binding to immobilized S protein, serial diluted K-874A (1 to 0.002 μg/ml) in 1% BSA/PBS was incubated, and K-874A which bound to S protein was detected by HRP-conjugated anti-VHH antibody (#128-035-232, Jackson Immuno Research). Between each incubation, wells were washed with PBST. To color, o-phenylenediamine dihydrochloride (p6662, Merck) was used as a substrate of HRP and reaction was stopped with 2 M H_2_SO_4_. Signaling was quantified by Ensight (Perkin Elmer).

### Immunofluorescence staining of SARS-CoV-2-infected cells

VeroE6/TMPRSS2 cells were infected SARS CoV-2 and fixed at 24 hr after infection. After treatment with 0.05% Triton-X and 3% BSA/PBS for permeabilization and blocking, cells were incubated with anti-NSP8 (5A10, GeneTex) or anti-dsRNA (rJ2, Merck) for 1 hr and subsequently with Alexa 568-conjugated goat anti-mouse IgG (A110301, Thermo Fisher Scientific) and Hoechest for an hour at room temperature. Images were taken by BZX800 (Keyence).

### Infection to alveolar organoid and VHH treatment

Human alveolar-derived organoids were established [20]. Briefly, 3D cultured organoid compound in Matrigel was treated with cell recovery solution to remove the cells from the Matrigel. The cells were infected with SARS-CoV-2 (MOI = 5) for 1 hr at 37°C, washed to remove unbound virus, and compounded into Matrigel. After the Matrigel solidified, VHH (15 μg/mL) was added or not. Culture supernatants were collected at each time point and preserved at -80°C.

### VHH treatment of SARS CoV-2-infected Syrian hamsters

2x10^3^ TCID_50_ of SARS-CoV-2 was nasally inoculated to 7 weeks age of Syrian hamsters. Then 3 mg of K-874A was nasally administrated just before, 1 day or 1 and 2 days after virus inoculation. Each hamster was weighed every day up to 4 days after inoculation. Lung tissues were homogenized at 4 days after inoculation, and RNA was extracted with Nucleospin RNA (TAKARA) for qRT-PCR.

### qRT-PCR for inflammatory cytokines

Primers and probes for hamsters were designed as reported [31]. The primers and probes are listed in Table 1. qRT-PCR experiments were performed on a Light Cycler 96 (Roche) with Luna Universal Probe One-Step RT-qPCR Kit (NEB), according to manufacturer’s instructions. Briefly, 50 ng of RNA was used for each reaction as template. The final concentrations of each test primer and probe set for target gene were 0.4 and 0.2 μM, respectively. The final concentrations of the internal control primer and probe set were 0.2 and 0.1 μM, respectively. Cycling conditions were as follows: 10 min at 50°C (initial reverse transcription), 20 sec at 95°C (inactivation and initial denaturation), and 40 cycles of 3 sec at 95°C, followed by 60 sec at 60°C per cycle.

**Table 1:** Detailed sequence information of the primers and probes for qRT-PCR.

		Forward primer (5’ to 3‘)	Reverse primer (5’ to 3‘)	Probe
Interleukin-1β	IL-1β	GGCTGATGCTCCCATTCG	CACGAGGCATTTCTGTTGTTCA	FAM-CAGCTGCACTGCAGGCTCCGAG-BHQ1
Interleukin 6	IL-6	CCTGAAAGCACTTGAAGAATTCC	GGTATGCTAAGGCACAGCACACT	FAM-AGAAGTCACCATGAGGTCTACTCGGCAAAA-BHQ1
Interleukin 10	IL-10	GTTGCCAAACCTTATCAGAAATGA	TTCTGGCCCGTGGTTCTCT	FAM-CAGTTTTACCTGGTAGAAGTGATGCCCCAGG-BHQ1
Tumor Necrosis Factor-α	TNF-α	GGAGTGGCTGAGCCATCGT	AGCTGGTTGTCTTTGAGAGACATG	FAM-CCAATGCCCTCCTGGCCAACG-BHQ1
β-actin	β-act	ACTGCCGCATCCTCTTCCT	TCGTTGCCAATGGTGATGAC	Cy5-CCTGGAGAAGAGCTATGAGCTGCCTGATG-BHQ2

### Cryo-electron microscopy

S protein trimers of SARS-CoV-2 (# SPN-C52H9, Acrobiosystems, 600 μg/mL) and K-874A (11 mg/mL) were mixed at 10:1 and kept at room temperature for 1 day. Aliquot (2.5 μL) of the sample was placed onto a holey-carbon copper grid (Quantifoil Micro Tools, R 1.2/1.3), previously glow-discharged in a plasma ion bombarder (PIB-10, Vacuum Device Inc.). The grid was then blotted (blotting time: 3.5 sec, blotting force: 7) and plunge-frozen at a condition of 95% humidity and 4°C by using a Vitrobot Mark IV (Thermo Fisher Scientific). The cryo-EM data were acquired with a Titan Krios at 300 kV (Thermo Fisher Scientific) and a Gatan K3 camera (Gatan) at a nominal magnification of 64,000, corresponding to 1.11Å per pixel on specimen. Each micrograph was recorded as a movie of 58 frames at the total dose of approximately 40 electrons per Å^2^. A GIF-quantum energy filter (Gatan) was used with a slit width of 20 eV to remove inelastically scattered electrons. Individual movies were subjected to per-frame drift correction by MotionCor2 [32]. The contrast transfer function parameters of each micrograph were estimated using CTFFIND4 [33] and the following 2D and 3D classification, 3D refinement, and local resolution calculation were performed with RELION3.1 software [34]. A total of 2,252,622 particles were automatically selected from 6,552 micrographs. The selected particles were divided into three groups and sorted by 2D classification to accelerate computation speed. The sorted particles were further divided into two classes by 3D classification. One is the structure in which K-874A binds to both RBD and NTD (type 1), and the other is the structure in which K-874A bind to only RBD (type 2). The ratio of type 1 and type 2 particles was approximately 9:1. In the case of type 1 particles, 3D classification was further performed against all particles classified as type 1, and 3D refinement was performed with only selected good classes. The final 3D reconstruction was computed with 115,297 particles. The global resolution of the final map was estimated to be 3.9Å (gold standard FSC criterion) by imposing C3 symmetry, but the external regions including K-874A, RBD, and NTD were too much noisy. Therefore, the external regions including K-874A, RBD, and NTD were individually extracted from symmetry-expanded particles, and focused refinement was performed. Using the “relion_particle_symmetry_expand” command [34], the regions including K-874A, RBD, and NTD in each particle image were independently expanded, and the generated 3 × 115,297 (345,891) sub-particle images were used for the 3D reconstruction. Next, to make a focused mask, the density including K-874A, RBD, and NTD was extracted from the final type 1 map using UCSF Chimera [35]. The extracted density was used to generate the mask using “Mask creation” command in RELION 3.1. The images containing K-874A, RBD, and NTD were extracted from the symmetry-expanded images with the prepared mask using “Particle subtraction” command in RELION3.1. These masked sub-particles were subjected to 3D classification without shift and rotation, and the sub-particle images (143,647 particles) of the selected good classes were used for 3D refinement by imposing C1 symmetry. The resolution of the subtracted map was estimated to be 5.0 Å based on a gold standard FSC. On the other hand, all type 2 particles from three groups were merged and subjected to 3D classification again. Then, selected good classes were subjected to 3D refinement. The final 3D map was reconstructed from 51,305 particles. The global resolution of the final map was estimated to be 4.4Å (gold standard FSC criterion) by imposing C3 symmetry, but the external region including K-874A, RBD, and NTD was too much noisy as the same as the type 1 particle. Therefore, the region, including K-874A, RBD, and NTD, was subjected to focused refinement in the same way as the type 1 particle. The resolution of the subtracted map was estimated to be 5.0Å, based on a gold standard FSC. The entire procedure and the processing data are summarized in S2 Fig and S1 Table.

The cryo-EM images of S protein trimer without K-874A were collected with a JEM2200FS electron microscope (JEOL) using a side-entry Gatan 626 cryo-specimen holder (Gatan). For image formation, a field-emission gun operated at 200 kV and an in-column (Omega-type) energy filter operating in zero-energy-loss mode with a slit width of 20 eV were used. Images of the frozen hydrated particles were recorded on a direct-detector CMOS camera (DE20, Direct Electron, LP) at a microscope magnification of 40,000×, corresponding to 1.422Å per pixel on the specimen. Using a low-dose method, the total electron dose on the specimen was about 20 electrons per Å^2^ for a 3-sec exposure. The single particle analysis was performed with RELION3.1. The 3D map reconstructed from 14,235 particles was estimated to be 6.9Å, based on a gold standard FSC. The entire procedure and the processing data are summarized in S1 Fig and S1 Table.

### Model building

The SWISS-MODEL [36] server was used to generate homology models of K-874A and S protein monomer of SARS-CoV-2 (SPN-C52H9) using the atomic models of VHH-72 (PDB ID: 6WAQ) and 2019-nCoV S protein (PDB ID: 6VSB) as templates, respectively. The amino acid sequence comparisons between SPN-C52H9 and 6VSB and between K-874A and VHH-72 are shown in S6 and S7 Figs. Multiple-sequence alignments of these proteins were performed using the PROMALS3D program [37]. The homology models were manually modified in the individual maps using COOT [38] and refined using PHENIX [39]. Type 1 and Type 2 structures consisting of the K-874A S protein trimer showed almost similar interactions with the same amino acid residues between K-874A and RBD, although the models were built independently. The amino acid residues in Type 1 and Type 2 are indicated in Figs 3C and S4C. The model statistics are summarized in S1 Table.

## Supporting information

S1 Table Cryo-EM data collection, image processing and validation statistics. (TIF)

S1 Fig Workflow for the cryo-EM analysis of the S protein trimer of SARS-CoV-2. Gold-standard Fourier shell correlation (FSC) curves show the global resolutions of the entire complex of the S protein trimer with the solid lines and the resolution of the focused refinement maps only, including RBD and NTD.
(TIF)

S2 Fig Workflow for the cryo-EM analysis of the S protein trimer of SARS-CoV-2 with K-874. Type 1 cryo-EM structure showed that K-874A bound to both RBD and NTD, and approximately 90% of the particles were classified into this structure. The remaining approximately 10% of particles was classified into the Type 2 cryo-EM structure, where took upward state and K-874A bound to RBD only, not NTD. The focused refinement was performed in the regions RBD, NTD, and K-874A for Types 1 and 2 structures. Gold-standard Fourier shell correlation (FSC) curves show the global resolutions of the entire complex of the S protein trimer and K-874A with the solid lines and the resolution of the focused refinement maps only, including RBD, NTD, and K-874A with dotted lines.
(TIF)

S3 Fig Cryo-EM maps and the fitted atomic model of the recombinant S trimer and the complex with K-874A. (**A-C**) Side views of the cryo-EM maps of the S protein trimer (A), type 1 structure (B) and type 2 structure (C) of the complex withK-874A. (B) and (C) are composite maps. (**D-F**) The fitted atomic models of the S protein monomer (D) or the complex with K-874A ((E) type 1 structure; (F) type2 structure). The NTD, RBD and K-874 are colored blue, green and magenta, respectively. (**G-I**) The atomic models of S protein trimer (G) or the complexes with K-874A (H, I) fitted into the maps. Scale bar equals 50Å.
(TIF)

S4 Fig Interaction of S protein trimer and K-874A VHHs in the minor structure, type 2. (**A**) A ribbon model of S protein trimer and K-874A. The coloring follows the standard designation of K-874A (magenta), NTD (blue) and RBD (green). (**B**) An enlarged view of RBD, NTD and K-874A in (A). (**C**) A view of the structure in (B) rotated 90 degrees. Residues that interact with ACE2 are shown in yellow. N501 and E484 (cyan) are amino acid residues frequently mutated.
(TIF)

S5 Fig Conformational changes in the S protein by K-874A binding. Light green and light blue are the positions of RBD and NTD of S protein when K-874A does not bind. (A) Type 1 structure of the complex with K-874A (B) Type 2 structure of the complex with K-874A. In the type 1 structure (approximately 90% of the S protein and K-874A complex), the K-874A bound moved the RBD sideways and the NTD down. In the case of type 2 structure (approximately 10% of the S protein and K-874A complex), the K-874A bound to the upward state of the RBD and the NTD did not interact directly with K-874 but moved slightly aside.
(TIF)

S6 Fig Alignment of the amino acid sequences of the two SARS-CoV-2 S protein. The secondary-structural elements are indicated over the sequences as a spiral (α-helix) or an arrow (β-sheet). The NTD and the RBD are colored by blue and green, respectively. Letters on a red background indicate identical amino acids. Figure is drawn by ESPript [40].
(TIF)

S7 Fig Alignment of the amino acid sequences of K-874A and VHH-72. The secondary-structural elements are indicated over the sequences as a spiral (α-helix) or an arrow (β-sheet). Letters on a red background indicate identical amino acids. Figure is drawn by ESPript [40].
(TIF)

S8 Fig K-874A VHH neutralizes B.1.1.7 variant but no other variants. (**A**) RNA copies in the culture supernatant at 0 or 2 days post-infection with or without K-874A. Calu3 cells were infected with each SARS-CoV-2 variant for 1 hr. After infection, cells were washed twice with fresh medium and cultured with or without 150 μg/ml of K-874A for 2 days. RNA copies at day 2 were measured by qRT-PCR. Wuhan, KUH003 (D614G) and B.1.1.7 (Alpha) were slightly reduced in K-874A-treated, but B.1.351 (Beta), P.1 (Gamma) and B.1.617.2 (Delta) were not. (**B**) RNA copies in each culture supernatant were adjusted to 10,000 copies based on qRT-PCR results in (A), and each variant with or without K-874A was infected to VeroE6/TMPRSS2 cells to determine TCID_50_. A significant difference between with or without K-874A was observed in culture supernatant with Wuhan, KUH003 (D614G) and B.1.1.7 (Alpha). (N = 5, * P<0.05, Welch’s t-test) (**C**) ELISA data showing direct binding of recombinant S protein from each variant and K-874A. Immobilized K-874A was incubated with Rho1D4-tagged recombinant S protein from each variant and detected by anti-rhodopsin IgG. K-874A bound to Wuhan, and B.1.1.7 (Alpha) but not others.
(TIF)

S9 Fig Different dose of VHH treatment to infected Syrian hamsters. (**A**) Time schedule for inoculation and K-874A administration to Syrian hamsters. (**B)** Weight changes in K-874A-treated and -untreated hamsters after SARS-CoV-2 infection as indicated. Weight at Day 0 is 100%. **(C)** Amounts of viral RNA in the lung homogenates at Day 4 were determined by qRT-PCR. (**D**) qRT-PCR results showing inflammatory cytokine expression. Expression levels of IL-6, IL-10, TNF-α and IL-1β were assessed in lung homogenates collected at Day 4. (N = 3, *P<0.05, **P<0.01, Dunnett’s multiple comparison test)
(TIF)

S10 Fig **Schematic diagram indicating K-874A binding domain (A) and ACE2-blocking VHHs binding domain (B).** Trimer of S proteins, ACE2 and VHHs were depicted and ACE2 binding domain of RBD was depicted in red. K-874A binds NTD and RBD, while previous reported VHHs binds the surface of ACE2 binding interface.
(TIF)
