# Supplementary figures and images for: Nasal delivery of single-domain antibody improves symptoms of SARS-CoV-2 infection in an animal model
Source: PLoS Pathog. 2021 Oct 14;17(10):e1009542. doi: 10.1371/journal.ppat.1009542 (PMC8516304; doi:10.1371/journal.ppat.1009542)

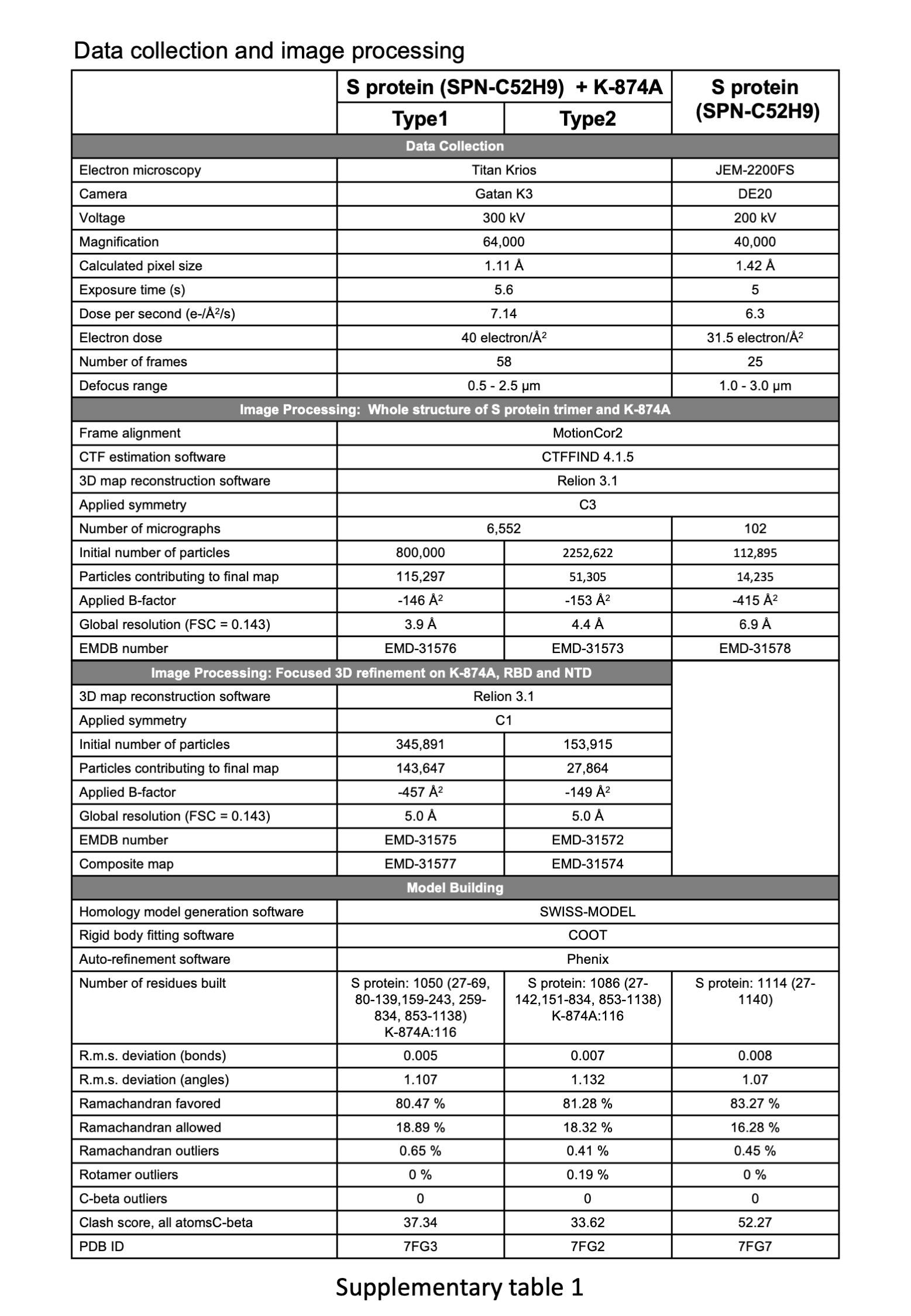

Supplement: S1 Table — (TIF) [file ppat.1009542.s001.tif]

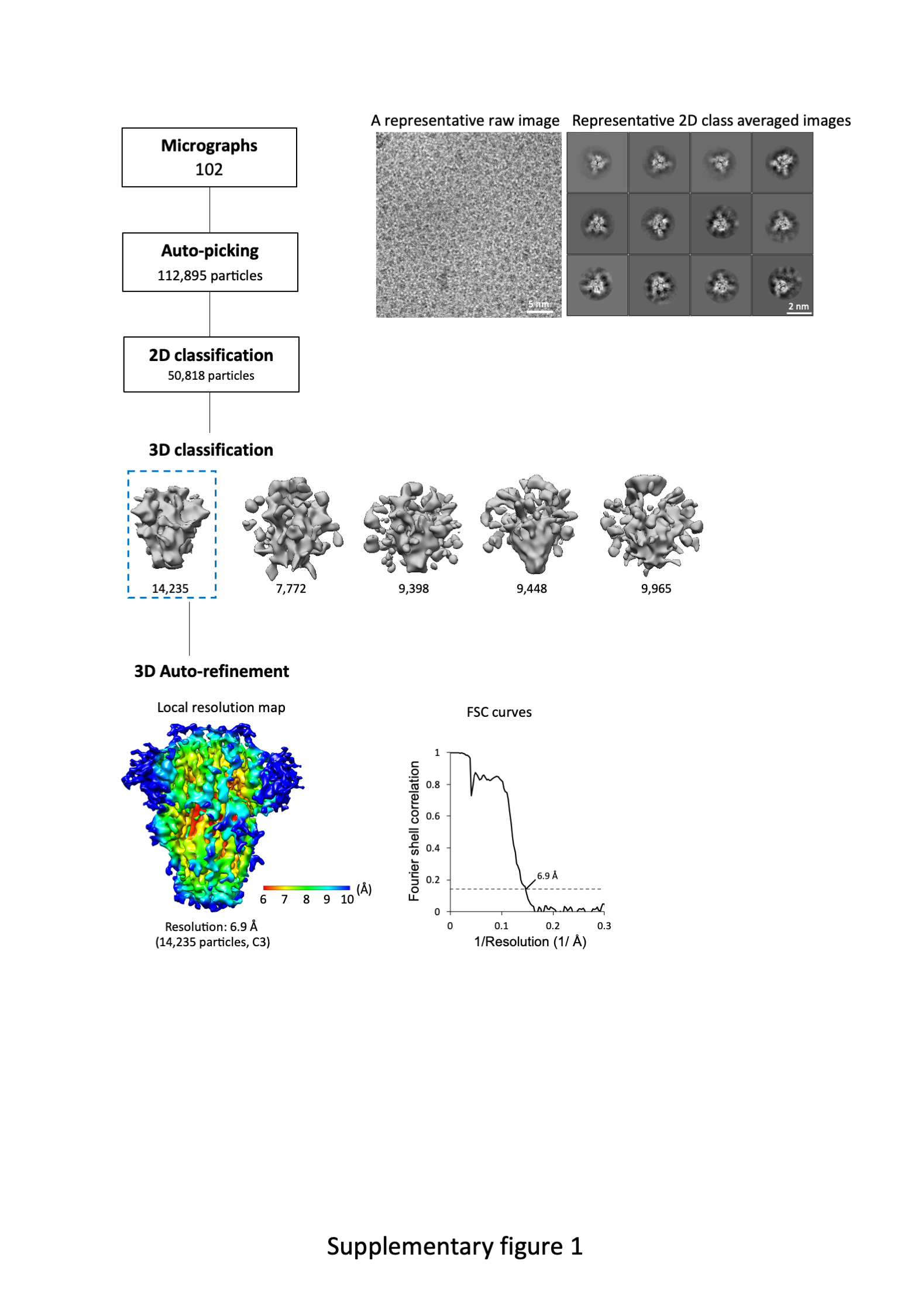

Supplement: S1 Fig — Gold-standard Fourier shell correlation (FSC) curves show the global resolutions of the entire complex of the S protein trimer with the solid lines and the resolution of the focused refinement maps only, including RBD and NTD. (TIF) [file ppat.1009542.s002.tif]

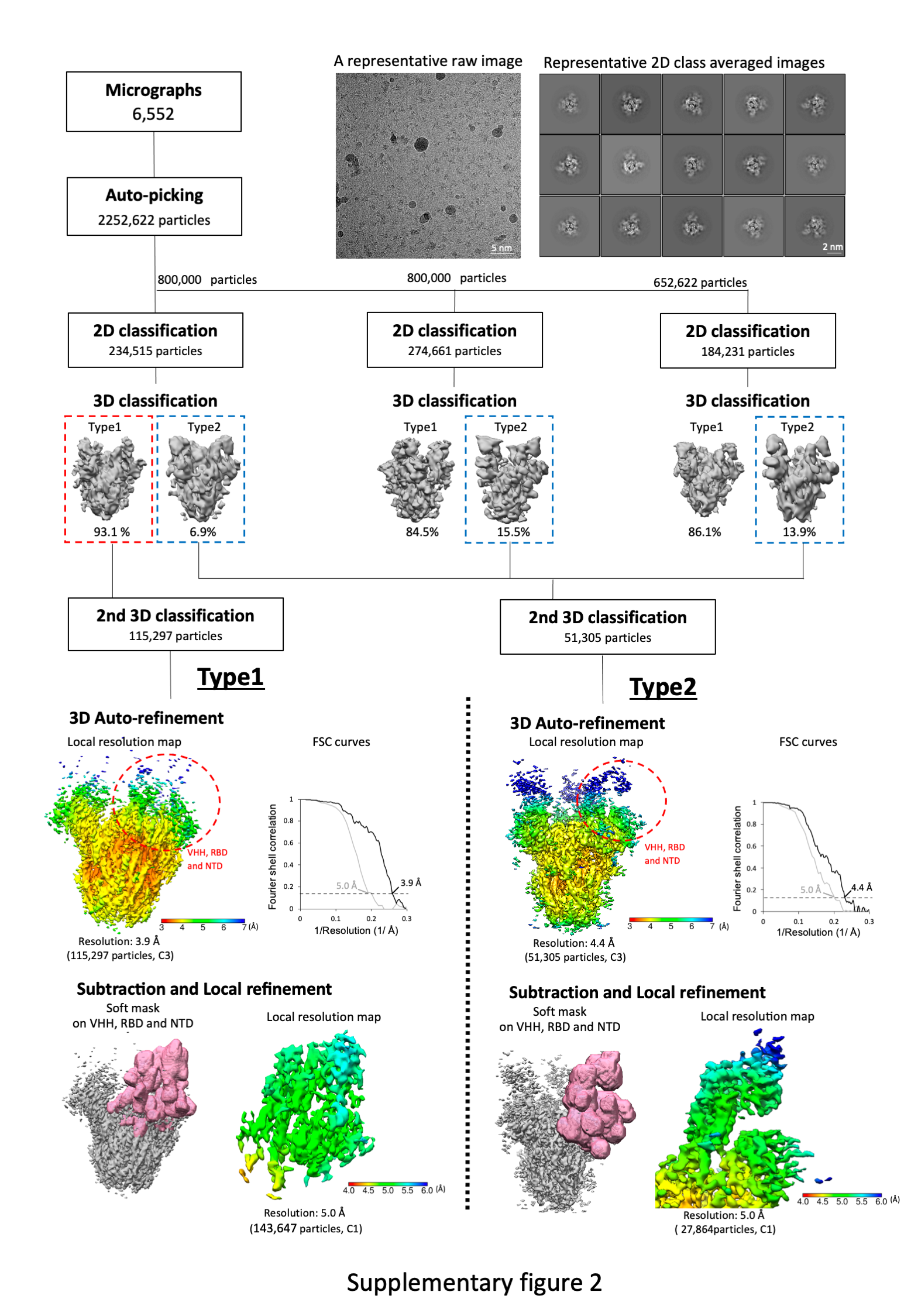

Supplement: S2 Fig — Type 1 cryo-EM structure showed that K-874A bound to both RBD and NTD, and approximately 90% of the particles were classified into this structure. The remaining approximately 10% of particles was classified into the Type 2 cryo-EM structure, where took upward state and K-874A bound to RBD only, not NTD. The focused refinement was performed in the regions RBD, NTD, and K-874A for Types 1 and 2 structures. Gold-standard Fourier shell correlation (FSC) curves show the global resolutions of the entire complex of the S protein trimer and K-874A with the solid lines and the resolution of the focused refinement maps only, including RBD, NTD, and K-874A with dotted lines. (TIF) [file ppat.1009542.s003.tif]

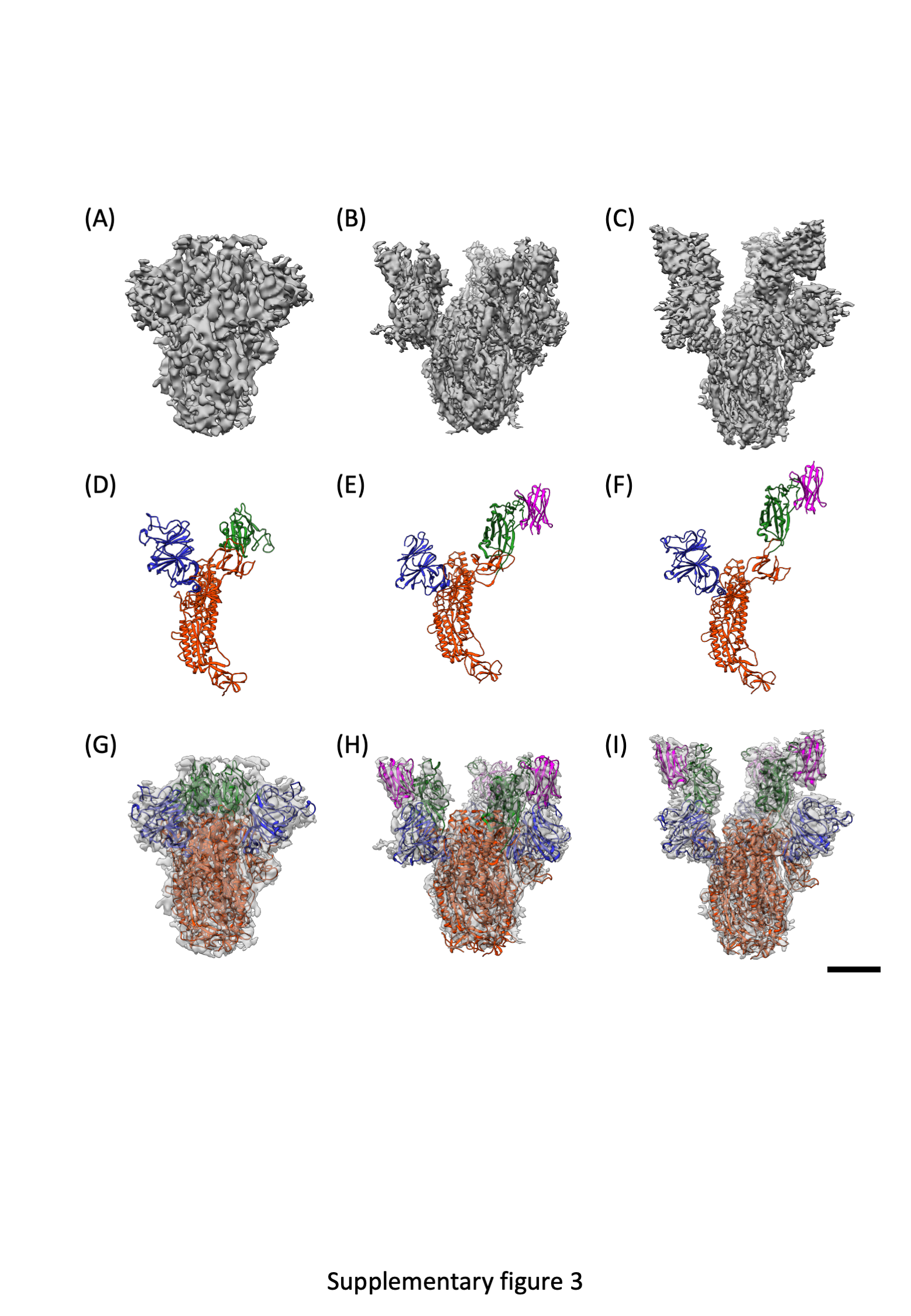

Supplement: S3 Fig — (A-C) Side views of the cryo-EM maps of the S protein trimer (A), type 1 structure (B) and type 2 structure (C) of the complex withK-874A. (B) and (C) are composite maps. (D-F) The fitted atomic models of the S protein monomer (D) or the complex with K-874A ((E) type 1 structure; (F) type2 structure). The NTD, RBD and K-874 are colored blue, green and magenta, respectively. (G-I) The atomic models of S protein trimer (G) or the complexes with K-874A (H, I) fitted into the maps. Scale bar equals 50Å. (TIF) [file ppat.1009542.s004.tif]

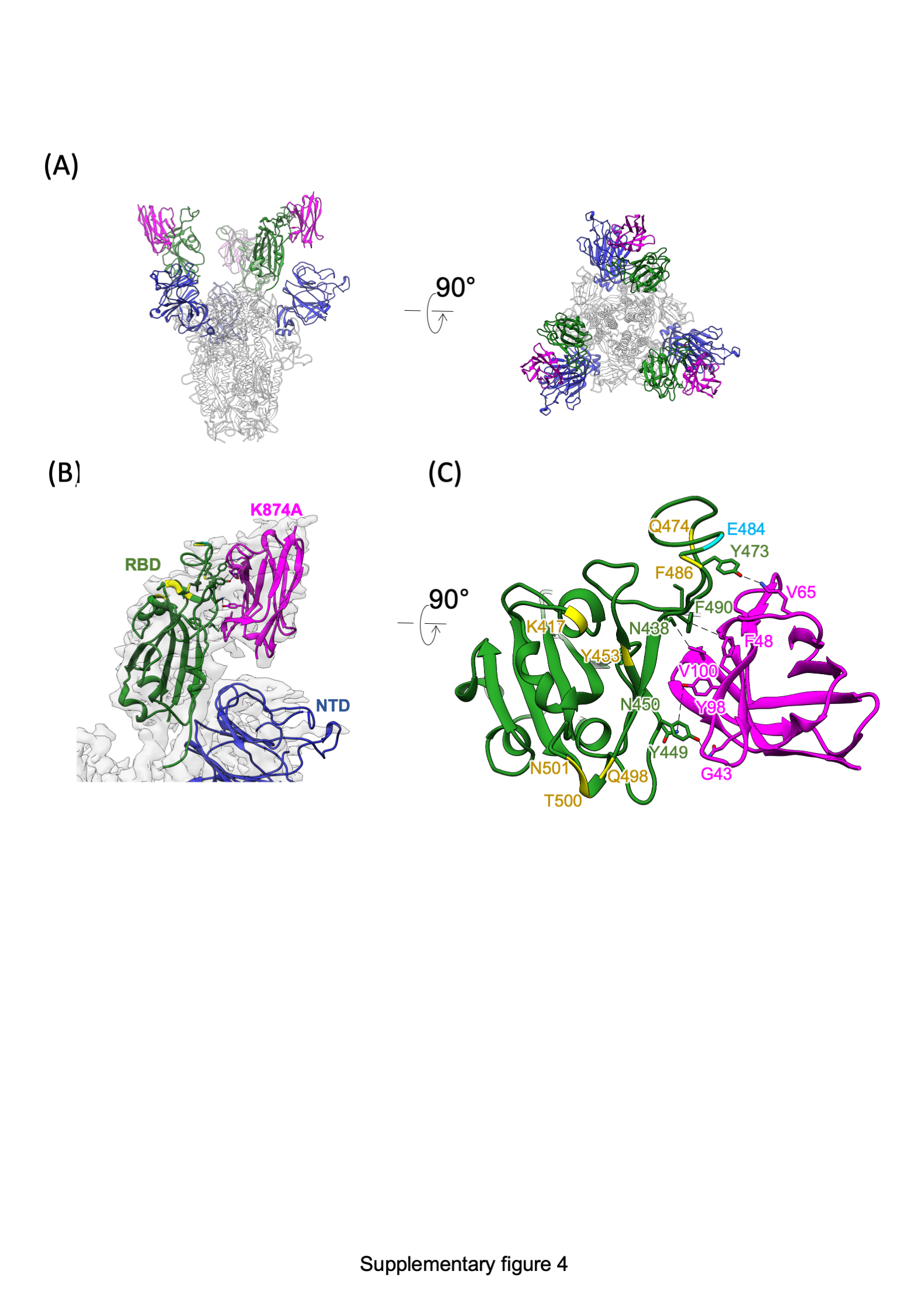

Supplement: S4 Fig — (A) A ribbon model of S protein trimer and K-874A. The coloring follows the standard designation of K-874A (magenta), NTD (blue) and RBD (green). (B) An enlarged view of RBD, NTD and K-874A in (A). (C) A view of the structure in (B) rotated 90 degrees. Residues that interact with ACE2 are shown in yellow. N501 and E484 (cyan) are amino acid residues frequently mutated. (TIF) [file ppat.1009542.s005.tif]

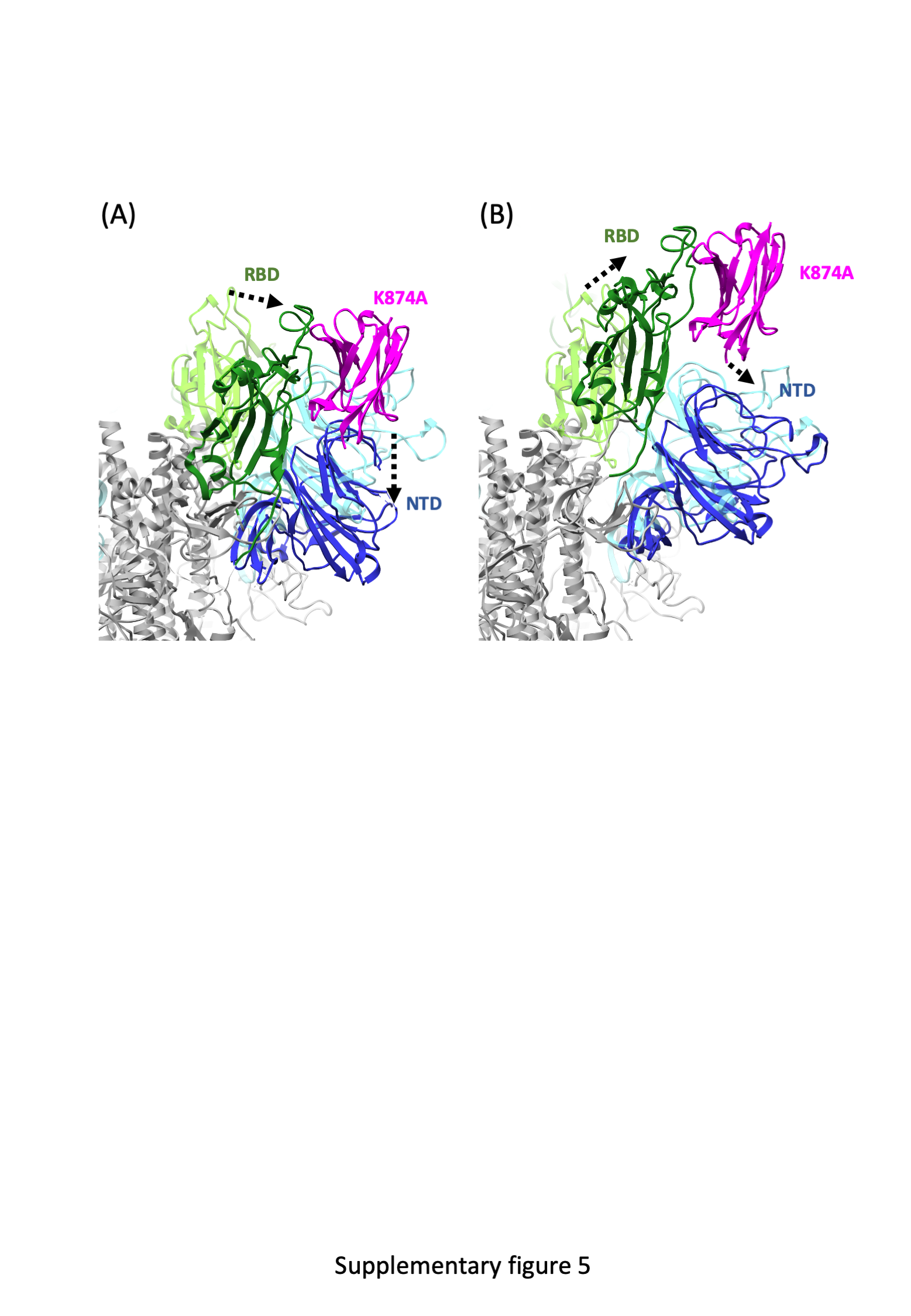

Supplement: S5 Fig — Light green and light blue are the positions of RBD and NTD of S protein when K-874A does not bind. (A) Type 1 structure of the complex with K-874A (B) Type 2 structure of the complex with K-874A. In the type 1 structure (approximately 90% of the S protein and K-874A complex), the K-874A bound moved the RBD sideways and the NTD down. In the case of type 2 structure (approximately 10% of the S protein and K-874A complex), the K-874A bound to the upward state of the RBD and the NTD did not interact directly with K-874 but moved slightly aside. (TIF) [file ppat.1009542.s006.tif]

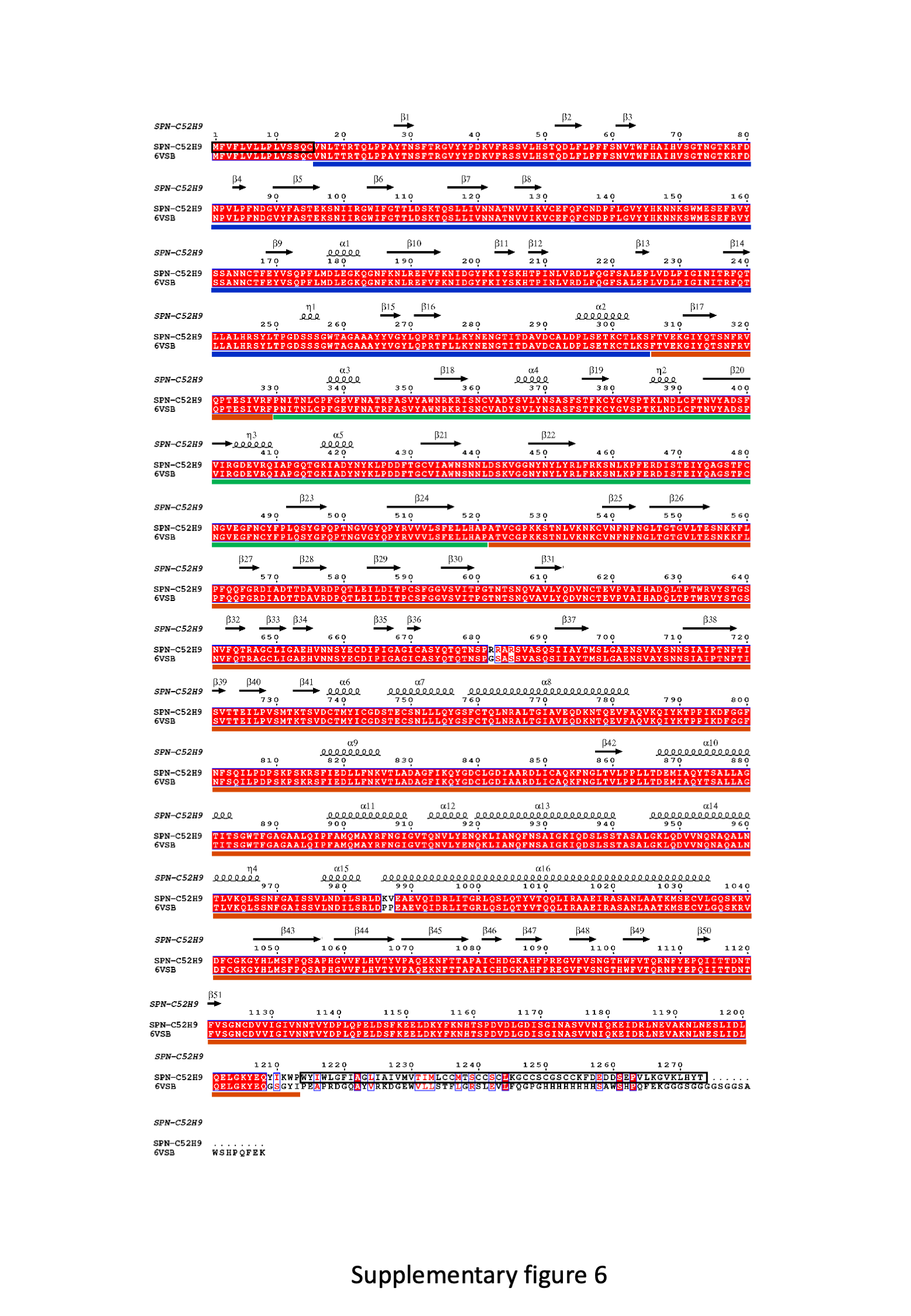

Supplement: S6 Fig — The secondary-structural elements are indicated over the sequences as a spiral (α-helix) or an arrow (β-sheet). The NTD and the RBD are colored by blue and green, respectively. Letters on a red background indicate identical amino acids. Figure is drawn by ESPript [40]. (TIF) [file ppat.1009542.s007.tif]

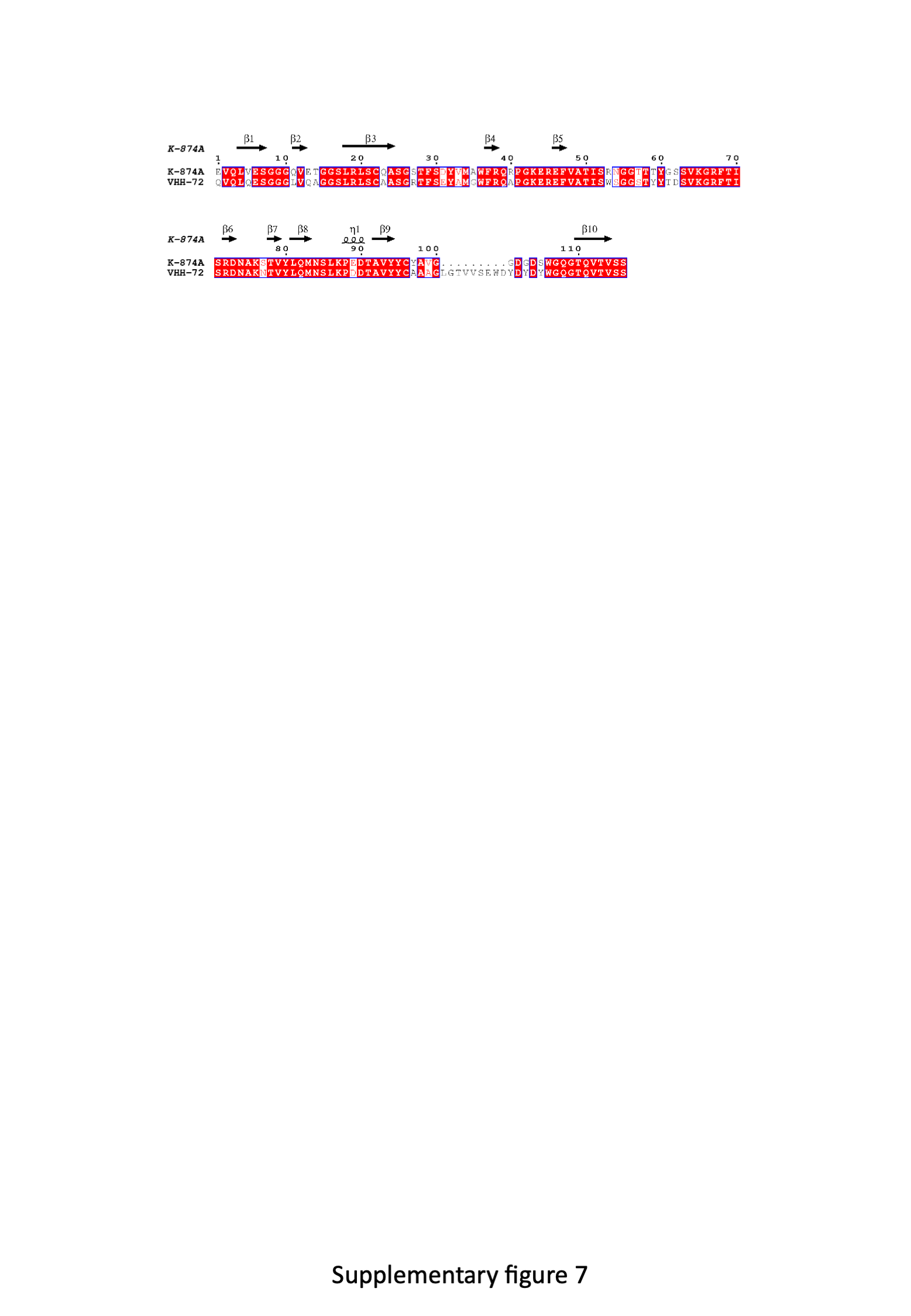

Supplement: S7 Fig — The secondary-structural elements are indicated over the sequences as a spiral (α-helix) or an arrow (β-sheet). Letters on a red background indicate identical amino acids. Figure is drawn by ESPript [40]. (TIF) [file ppat.1009542.s008.tif]

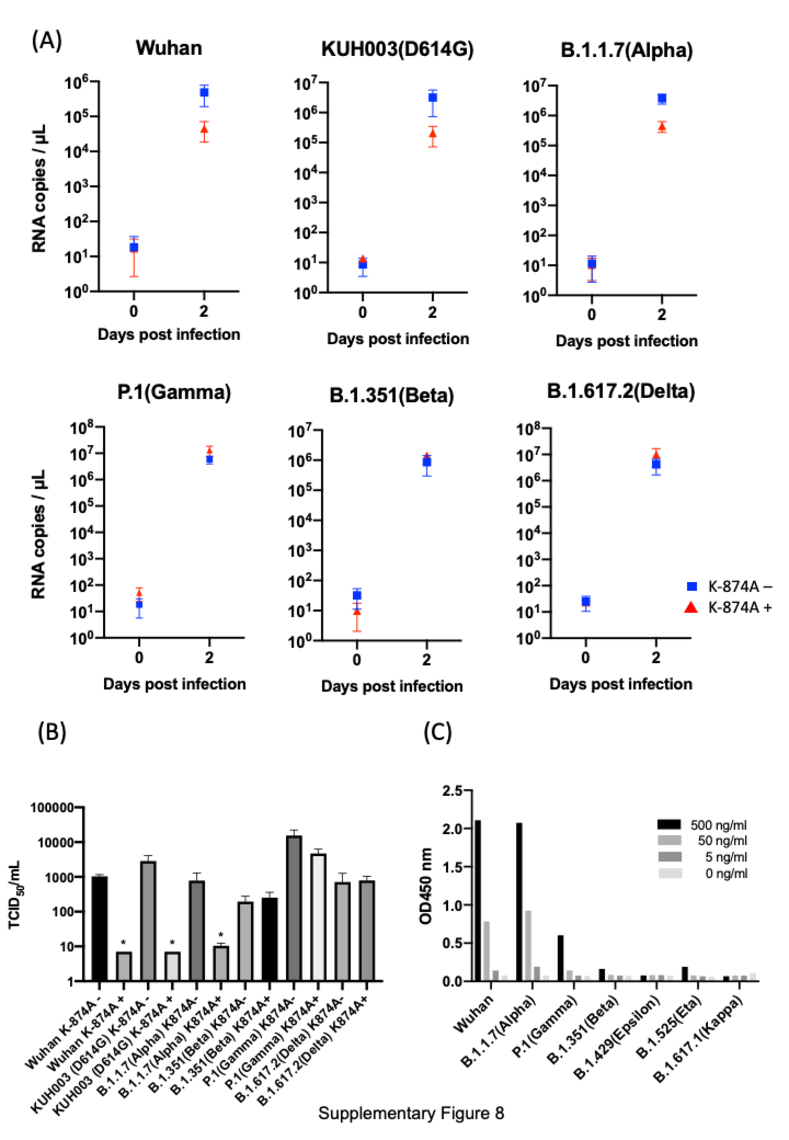

Supplement: S8 Fig — (A) RNA copies in the culture supernatant at 0 or 2 days post-infection with or without K-874A. Calu3 cells were infected with each SARS-CoV-2 variant for 1 hr. After infection, cells were washed twice with fresh medium and cultured with or without 150 μg/ml of K-874A for 2 days. RNA copies at day 2 were measured by qRT-PCR. Wuhan, KUH003 (D614G) and B.1.1.7 (Alpha) were slightly reduced in K-874A-treated, but B.1.351 (Beta), P.1 (Gamma) and B.1.617.2 (Delta) were not. (B) RNA copies in each culture supernatant were adjusted to 10,000 copies based on qRT-PCR results in (A), and each variant with or without K-874A was infected to VeroE6/TMPRSS2 cells to determine TCID50. A significant difference between with or without K-874A was observed in culture supernatant with Wuhan, KUH003 (D614G) and B.1.1.7 (Alpha). (N = 5, * P<0.05, Welch’s t-test) (C) ELISA data showing direct binding of recombinant S protein from each variant and K-874A. Immobilized K-874A was incubated with Rho1D4-tagged recombinant S protein from each variant and detected by anti-rhodopsin IgG. K-874A bound to Wuhan, and B.1.1.7 (Alpha) but not others. (TIF) [file ppat.1009542.s009.tif]

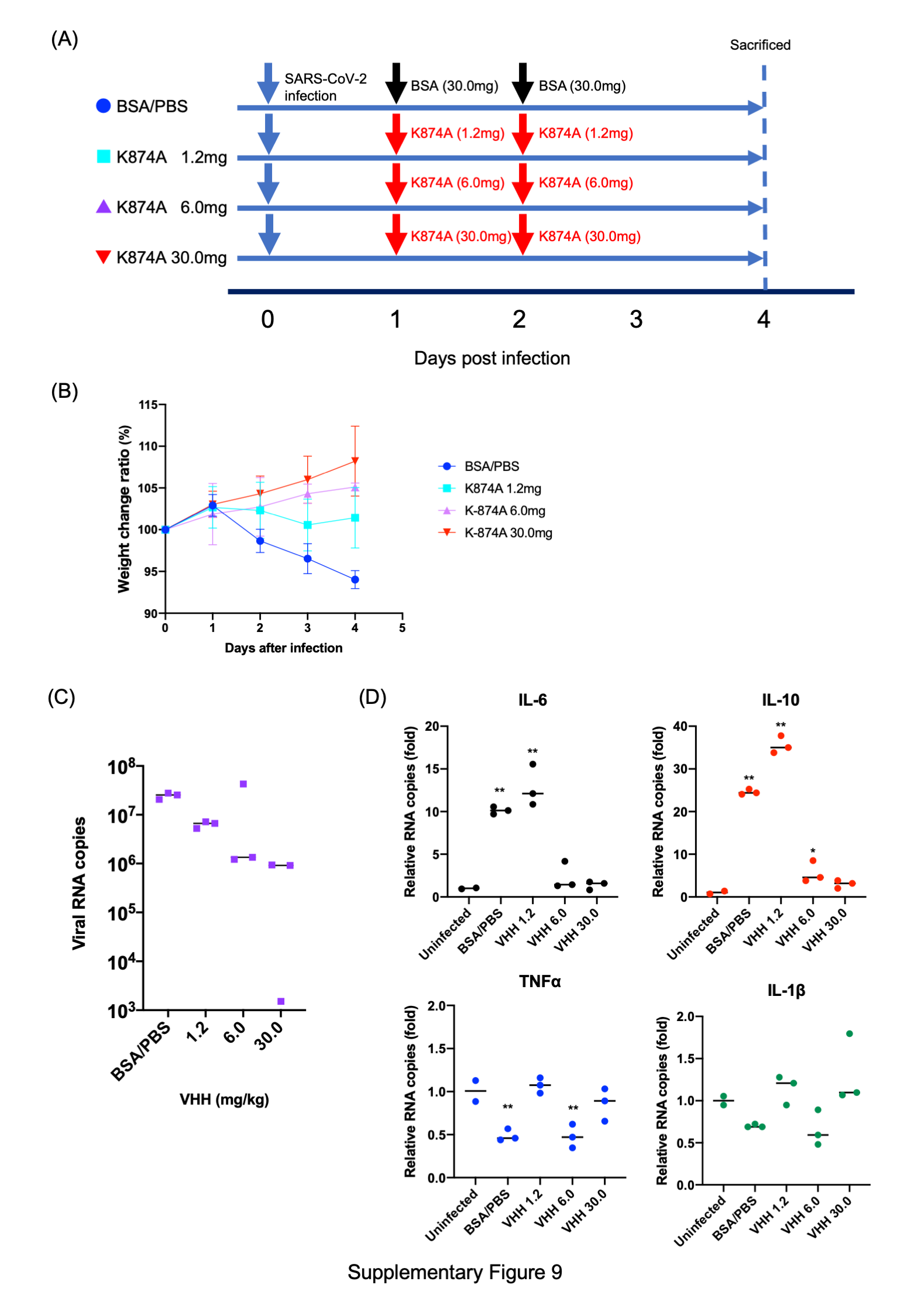

Supplement: S9 Fig — (A) Time schedule for inoculation and K-874A administration to Syrian hamsters. (B) Weight changes in K-874A-treated and -untreated hamsters after SARS-CoV-2 infection as indicated. Weight at Day 0 is 100%. (C) Amounts of viral RNA in the lung homogenates at Day 4 were determined by qRT-PCR. (D) qRT-PCR results showing inflammatory cytokine expression. Expression levels of IL-6, IL-10, TNF-α and IL-1β were assessed in lung homogenates collected at Day 4. (N = 3, *P<0.05, **P<0.01, Dunnett’s multiple comparison test) (TIF) [file ppat.1009542.s010.tif]

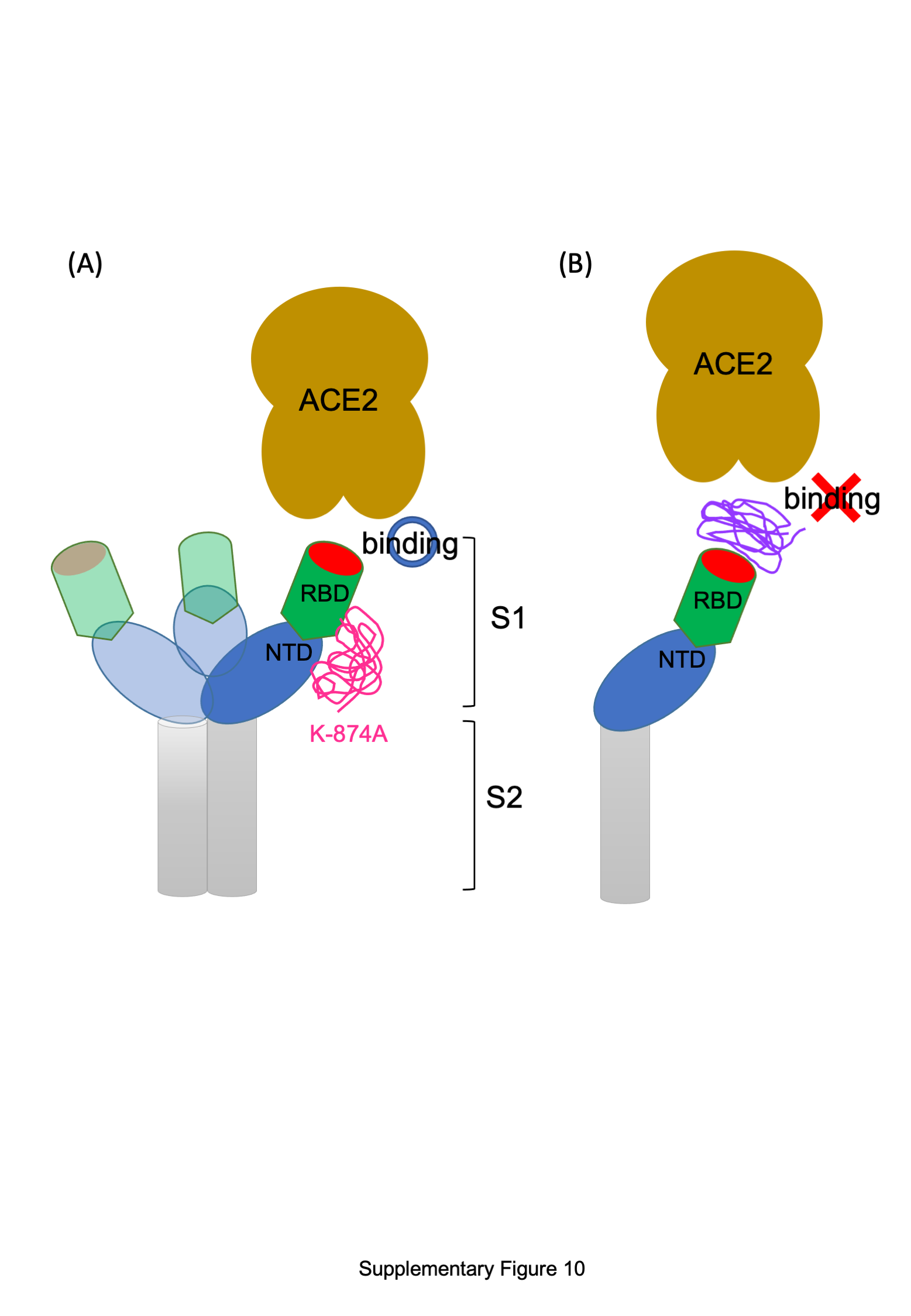

Supplement: S10 Fig — Schematic diagram indicating K-874A binding domain (A) and ACE2-blocking VHHs binding domain (B). Trimer of S proteins, ACE2 and VHHs were depicted and ACE2 binding domain of RBD was depicted in red. K-874A binds NTD and RBD, while previous reported VHHs binds the surface of ACE2 binding interface. (TIF) [file ppat.1009542.s011.tif]
